# Exposure to antibiotics and risk of latent autoimmune diabetes in adults and type 2 diabetes: results from a Swedish case–control study (ESTRID) and the Norwegian HUNT study

**DOI:** 10.1007/s00125-024-06302-5

**Published:** 2024-10-28

**Authors:** Jessica Edstorp, Marios Rossides, Emma Ahlqvist, Lars Alfredsson, Johan Askling, Daniela Di Giuseppe, Valdemar Grill, Elin P. Sorgjerd, Tiinamaija Tuomi, Bjørn O. Åsvold, Sofia Carlsson

**Affiliations:** 1 Institute of Environmental Medicine, Karolinska Institutet, Stockholm, Sweden; 2 Department of Respiratory Medicine and Allergy, Theme Inflammation and Ageing, Karolinska University Hospital, Stockholm, Sweden; 3 Department of Clinical Sciences in Malmö, Lund University Diabetes Centre, Lund University, Malmö, Sweden; 4 Center for Occupational and Environmental Medicine, Region Stockholm, Stockholm, Sweden; 5 Clinical Epidemiology Division, Department of Medicine Solna, Karolinska Institutet, Stockholm, Sweden; 6 Department of Clinical and Molecular Medicine, Norwegian University of Science and Technology, Trondheim, Norway; 7 HUNT Research Center, Department of Public Health and Nursing, Norwegian University of Science and Technology, Levanger, Norway; 8 Department of Endocrinology, Clinic of Medicine, St Olavs Hospital, Trondheim, Norway; 9 Institute for Molecular Medicine Finland, Helsinki University, Helsinki, Finland; 10 Division of Endocrinology, Abdominal Center, Helsinki University Hospital, Helsinki, Finland; 11 Research Program for Diabetes and Obesity, University of Helsinki, Helsinki, Finland; 12 Folkhälsan Research Center, Helsinki, Finland; 13 HUNT Center for Molecular and Clinical Epidemiology, Department of Public Health and Nursing, Norwegian University of Science and Technology, Trondheim, Norway

**Keywords:** Antibacterial agent, Case–control study, Diabetes mellitus, ESTRID, HUNT, LADA, Latent autoimmune diabetes in adults, Registries, Type 2 diabetes

## Abstract

**Aims/hypothesis:**

Some studies find an increased risk of type 1 diabetes in children exposed to antibiotics. We investigated if exposure to antibiotics increases the risk of latent autoimmune diabetes in adults (LADA) and type 2 diabetes.

**Methods:**

We used data from a Swedish case–control study (Epidemiological Study of Risk Factors for LADA and Type 2 Diabetes [ESTRID]: LADA, *n*=597; type 2 diabetes, *n*=2065; control participants matched on participation time, *n*=2386) and a case–control study nested within the Norwegian Trøndelag Health Study (HUNT) (*n*=82/1279/2050). Anatomical Therapeutic Chemical (ATC) codes indicating antibiotic dispensations were retrieved from the Swedish National Prescribed Drug Register and Norwegian Prescription Database. Multivariable adjusted ORs with 95% CIs were estimated by conditional logistic regression and pooled using fixed-effects inverse-variance weighting.

**Results:**

We observed no increased risk of LADA with exposure to antibiotics up to 1 year (OR_pooled_ 1.15, 95% CI 0.93, 1.41) or 1–5 years (OR_pooled_ 0.98, 95% CI 0.80, 1.20) prior to diagnosis/matching for one or more vs no dispensation of any type of antibiotic. An increased risk was observed for one or more vs no dispensations of narrow-spectrum antibiotics, but not broad-spectrum antibiotics, 6–10 years prior to LADA diagnosis (OR_pooled_ 1.39, 95% CI 1.01, 1.91), which was driven by the Swedish data. There was little evidence of an increased risk of type 2 diabetes associated with antibiotic exposure 1–10 years prior to diagnosis.

**Conclusions/interpretation:**

We found no evidence that exposure to broad-spectrum antibiotics up to 10 years prior to diagnosis increases the risk of LADA. There was some indication of increased LADA risk with exposure to narrow-spectrum antibiotics, which warrants further investigation.

**Graphical Abstract:**

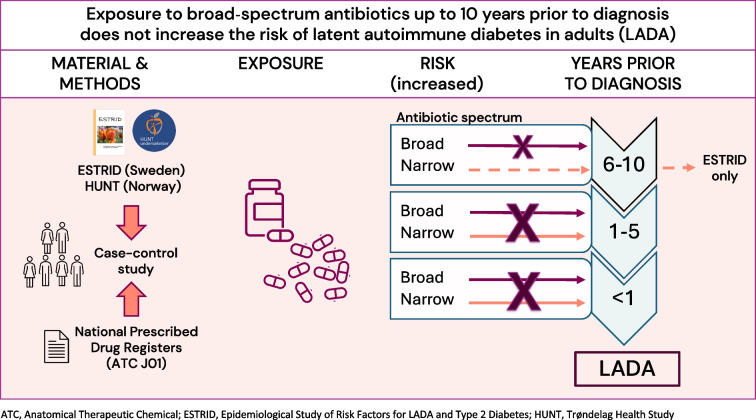

**Supplementary Information:**

The online version of this article (10.1007/s00125-024-06302-5) contains peer-reviewed but unedited supplementary material.


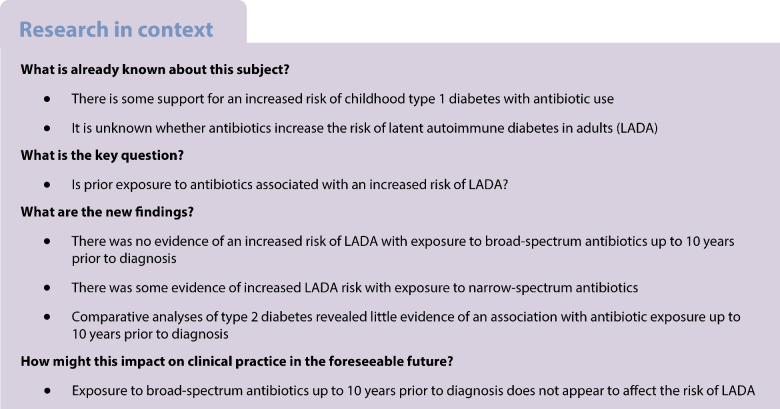


## Introduction

Latent autoimmune diabetes in adults (LADA) is a heterogeneous type of diabetes resembling type 1 diabetes in terms of genetic risk and autoimmunity, but is often mistaken for type 2 diabetes because of phenotypical similarities that encompass adiposity, insulin resistance and an adult onset [1, 2].

LADA is the most common form of autoimmune diabetes with onset in adulthood, yet little is known about risk factors beyond lifestyle risk factors that are shared with type 2 diabetes [3]. The phenotypical resemblance to type 2 diabetes implies that insulin resistance also plays a role in the pathogenesis of LADA. In contrast, it is not known what triggers the autoimmune reaction preceding LADA besides heritability. Antibiotics may be such a trigger, and an increased risk of childhood-onset type 1 diabetes with antibiotic use has been observed [4, 5], which in some studies is restricted to frequent use [6, 7] although other studies suggest no association [8, 9]. In type 2 diabetes, evidence indicates a higher risk associated with higher mean daily dose [10] higher frequency [11, 12] and longer duration [13] of antibiotic use.

We hypothesised that prior exposure to antibiotics is associated with LADA. Therefore, our aim was to conduct, to our knowledge, the first study investigating a link between LADA and prior exposure to antibiotics in adulthood and, further, to compare with results with those for type 2 diabetes. As LADA is a heterogeneous disease [2], we also examined whether an increased risk would be contingent on levels of GADA, which is the most common autoantibody in LADA. To obtain sufficiently precise estimates, we combined data from two studies with incident cases of LADA and type 2 diabetes.

## Methods

### The ESTRID study

#### Study population and design

**Fig. 1:**
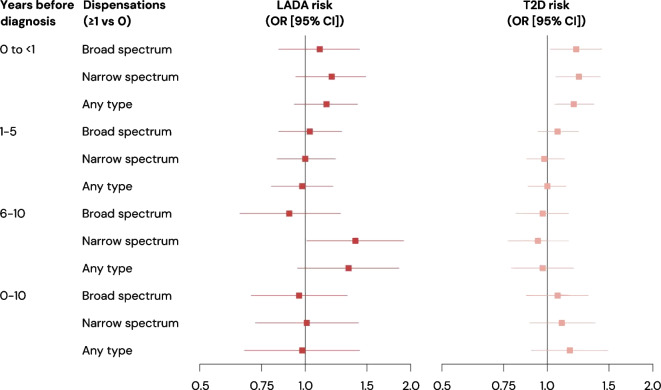
Pooled ORs (ESTRID and HUNT) with 95% CIs for the association between one or more vs no dispensations of antibiotics in different exposure windows and risk of LADA or type 2 diabetes (T2D)

The Epidemiological Study of Risk Factors for LADA and Type 2 Diabetes (ESTRID) is a Swedish, population-based, case–control study with incident cases of LADA and type 2 diabetes, nested within the All New Diabetics in Scania (ANDIS) register. Details on ANDIS and the study design are outlined in the Electronic Supplementary Material (ESM) Fig. 1 and ESM Methods. Sex was identified through each individual’s personal identity number. The proportions of men and women in ESTRID are fairly representative of the Swedish population, whereas people born abroad are under-represented. Participants provide health and lifestyle information through a detailed questionnaire and clinical information is available for cases through ANDIS. The analytical dataset included all incident cases of LADA (*n*=597) and type 2 diabetes (*n*=2065) along with control participants (*n*=2386) enrolled in ESTRID in 2010–2019. All participants provided written informed consent and the study was approved by the regional ethical review board in Stockholm (2018-1036-31, 2018-1036-32, 2023-06428-02).

#### Diabetes classification

Incident cases in ANDIS were classified as LADA or type 2 diabetes based on age at diagnosis (≥35 years) and analysis of GADA, measured by ELISA (RSR, UK, with sensitivity/specificity 0.84/0.98 [14]), and C-peptide levels (IMMULITE 2000, Siemens Healthcare Diagnostics Products, UK, or Cobas e601, Roche Diagnostics, Germany). Individuals with LADA were GADA positive (≥10 U/ml) and had C-peptide levels of ≥0.2 nmol/l or ≥0.3 nmol/l (IMMULITE/Cobas). Individuals with type 2 diabetes were GADA negative and had C-peptide levels of >0.60 or >0.72 nmol/l (IMMULITE/Cobas). HOMA-IR and HOMA-B were calculated based on fasting C-peptide and glucose levels [15].

### The HUNT study

#### Study population and design

In the Nord-Trøndelag region of Norway, the entire population aged ≥20 years has been invited to participate in the Trøndelag Health Study (HUNT) on four occasions between 1984 and 2019. More women than men participated in the HUNT surveys, and the highest participation rates were among those aged 50–79 years [16]. Sex was determined based on Norwegian national identification numbers. Information on ethnicity was not collected in the HUNT surveys. Participants respond to questionnaires and undergo clinical examination and blood sampling. We conducted a nested case–control study among cases and matched control participants in HUNT4, as well as HUNT3 if the year of diagnosis of cases was ≥2006 (prescription data available in Norway from 2004). The analytical sample included incident cases of LADA (*n*=82) and type 2 diabetes (*n*=1279) and matched control participants (*n*=2050). Details of the study design are outlined in ESM Methods. The Norwegian Data Protection Authority and the Regional Committee for Medical and Health Research Ethics approved the study (REK 140824) and all participants provided informed consent.

### Diabetes classification

Cases were identified through a questionnaire including the question ‘Have you had or do you have diabetes (yes/no)?’. GADA was assessed at a median of 5 years after diagnosis and used with self-reported age at diagnosis to determine diabetes type. GADA analysis was carried out at the Hormone Laboratory, Oslo University Hospital, Norway, either by immunoprecipitation radioligand assay (Novo Nordisk, Denmark; HUNT3) or by ELISA (RSR; HUNT4). The sensitivity and specificity of the immunoprecipitation assay were 0.64/1.00 (Islet Autoantibody Standardization Program 2003) [17] and of the ELISA assay were 0.84/0.98 (Islet Autoantibody Standardization Program 2020; P. M. Thorsby, Hormone Laboratory, Oslo University Hospital, Oslo, Norway, personal communication). Cases (≥35 years) were classified as LADA if positive for GADA (≥0.08 antibody index) in HUNT3; ≥10 U/ml in HUNT4); otherwise, they were classified as type 2 diabetes. C-peptide data from the time of diagnosis were not available in HUNT.

### Register linkages

In ESTRID, we retrieved information on antibiotic dispensations (Anatomical Therapeutic Chemical [ATC] codes J01, A07AA09, J04AB02, P01AB01) from the National Prescribed Drug Register (NPDR) [18]. HUNT participants were linked to the Norwegian Prescription Database (NorPD) from which we retrieved information on antibiotic dispensations according to ATC code J01. These registers record all prescriptions dispensed at pharmacies since 2005 and 2004, respectively. Diagnoses for comorbidity adjustment (ESTRID only) were identified from the Swedish National Patient Register (NPR) [19] and the Scania Healthcare Register (SHR) [20].

### Antibiotic use

Antibiotics were categorised into broad or narrow spectrum or any type (broad and/or narrow spectrum) [21] (ESM Table 1). Phenoxymethylpenicillin (ATC code J01CE02) was the most common antibiotic dispensed in both ESTRID and HUNT. Dispensations (number and % of total dispensations) of the most dispensed antibiotics 1–5 years prior to the index date are listed in ESM Table 2. Exposure windows were defined as 0 to <1, 1–5, 6–10 and 0–10 years prior to the index date. In ESTRID, the index date was set to the date of diagnosis (cases) and date of participation (control participants). In HUNT, the index date for cases and matched control participants was set to the year of diabetes diagnosis of the cases. Within the exposure windows, the different types of antibiotics were analysed categorically and continuously as number of dispensed prescriptions (0, 1–2, 3–4, ≥5) or as exposure duration, that is, as consecutive days per longest course (0, 1–14, ≥15) or as cumulative exposure (0, 1–19, ≥20 days [20–49, ≥50 days in the 0–10 years exposure window]), corresponding to the total number of exposed days over the period of interest. Exposed days were calculated as dispensed quantity × defined daily dosage (DDD), which is ‘the assumed average maintenance dose per day for a drug used for its main indication in adults’ [22]. Exposed days were calculated only in ESTRID, as information to calculate this was lacking in HUNT.

### Covariates

The covariate selection is illustrated in a directed acyclic graph (ESM Fig. 2). Age at participation and sex were based on or derived from national population registers. BMI (kg/m^2^) was based on anthropometric measurements in HUNT and self-reported weight and height in ESTRID. Smoking status (never/former/current), physical activity level (four categories ranging from sedentary to high), education level (primary school/upper secondary school/university) and family history of diabetes (yes/no) were self-reported. The percentage of missing values of covariates was <2% in both ESTRID and HUNT. We calculated high-dimensional propensity scores (hd-PS) [23] in ESTRID based on ICD-10-coded diagnoses (https://icd.who.int/browse10/2019/en) and ATC-coded dispensations from the registers, to adjust for potential confounding from comorbidity (see ESM Methods).

### Statistical analysis

Two-sided *p* values, calculated using an unpaired Student’s *t* test for means (±SDs) of normally distributed variables, Kruskal–Wallis test for medians (IQRs) of non-normally distributed variables, and *χ*^2^ test for proportions, were used to assess differences in baseline characteristics between the study groups.

Within the exposure windows 0 to <1, 1–5, 6–10 and 0–10 years prior to the index date, the associations between antibiotic dispensations (one or more vs none; one to two, three to four or five or more vs none; and per dispensation) and LADA/type 2 diabetes were analysed by logistic regression conditioned on participation time (ESTRID) and index year and sex (HUNT), estimating ORs with 95% CIs. Model 1 was adjusted for age and sex (matching variable in HUNT), whereas model 2 was additionally adjusted for BMI, smoking status, physical activity level, education level and family history of diabetes. Imputation was performed on missing values of the covariates based on the median values, and an indicator variable for missingness was included in the models. Study-specific estimates were meta-analysed using fixed-effects inverse-variance weighting (OR_pooled_). All analyses were performed in SAS 9.4 (SAS Institute, USA). Results from the fully adjusted model (model 2) are presented.

### Sensitivity analyses

To account for comorbidity and residual confounding, we adjusted the analyses in ESTRID for age, sex, ethnicity (born in Sweden: yes/no) and a continuous hd-PS considering all other covariates in model 2 in addition to comorbidity and elevated blood glucose. In ESTRID, we could also analyse the association between consecutive and cumulative antibiotic exposure and risk of LADA or type 2 diabetes. We further distinguished between LADA cases with high (GADA_high_) and low (GADA_low_) GADA levels based on the median value due to the highly skewed distribution (ESM Figs 3 and 4). Finally, we performed the same analysis with dermatological drugs (ATC codes D01, D04, D05, D10) as a negative control exposure.

## Results

In total, 679 LADA cases, 3344 type 2 diabetes cases and 4436 control participants were included in the study (Table 1). Compared with type 2 diabetes cases, LADA cases were on average younger, were more likely to be female, had a lower BMI, were more often on insulin treatment, and had lower HOMA-B and HOMA-IR indices. Carrying a type 1 diabetes-associated high-risk genotype (HLA) was more common among LADA cases than among type 2 diabetes cases. Among participants in ESTRID and HUNT, 56% and 60%, respectively, had at least one antibiotic dispensation 1–5 years prior to the index date.

**Table 1:** Characteristics of the study populations

Characteristic	ESTRID				HUNT			
	Control	LADA	Type 2 diabetes	*p* value	Control	LADA	Type 2 diabetes	*p* value
Participants, *n*	2386	597	2065		2050	82	1279	
Men, %	47.3	53.1	60.2	0.002	48.8	48.8	57.7	0.114
Age at diagnosis,^a^ years	58.9 (13.8)	59.1 (12.3)	63.2 (10.4)	<0.0001	47.9 (14.3)	58.1 (10.7)	59.9 (9.9)	0.118
BMI, kg/m^2^	26.0 (4.2)	28.4 (5.6)	31.2 (5.4)	<0.0001	26.5 (4.1)	29.7 (4.6)	30.5 (4.4)	0.114
Insulin treatment^b^, %	–	40.0	5.9	<0.0001	–	40.3	7.9	<0.0001
C-peptide,^c^ nmol/l	–	0.72 (0.49–1.22)	1.20 (0.97–1.74)	<0.0001	–	1.06 (0.67–1.39)	1.11 (0.89–1.41)	0.764
HOMA-IR^d^	–	2.82 (1.83–4.44)	3.55 (2.73–4.76)	<0.0001	–	–	–	
HOMA-B^d^	–	40.5 (14.9–70.0)	71.1 (44.4–95.6)	<0.0001	–	–	–	
High-risk HLA^e^, *%*	–	60.3	31.1	<0.0001	29.5	55.1	26.4	<0.0001
Any antibiotic dispensation^f^, *n* (%)	1328 (55.7)	248 (49.4)	1120 (58.4)		831 (56.8)	44 (71.0)	831 (65.0)	

Data are mean ± SD or median (IQR) unless stated otherwise. *p* values are shown for the difference between LADA and type 2 diabetes

^a^Age at participation for control participants

^b^Current use of insulin

^c^C-peptide levels not from time of diagnosis in HUNT

^d^HOMA-IR and HOMA-B were calculated based on fasting plasma glucose and serum C-peptide levels at diagnosis

^e^High-risk genotypes defined as DR3/3, DR3/4, DR4/4 or haplotypes of DR4-DQ8 or DR3-DQ2 (ESTRID) or presence of at least one of the risk variants inferring either DR3-DQ2 or DR4-DQ8 (HUNT)

^f^Any dispensation of any type of antibiotic within 1–5 years prior to diagnosis/matching in those with full 1- to 5-year follow-up data in the registers

### Antibiotic exposure and LADA

No association between antibiotic dispensations and LADA was observed up to 5 years prior to diagnosis, irrespective of type of antibiotic (broad or narrow spectrum), number of dispensations or days of exposure. For example, OR_pooled_ for one or more vs no dispensations of any type of antibiotic were 1.15 (95% CI 0.93, 1.41) and 0.98 (95% CI 0.80, 1.20) in the exposure windows up to 1 year and 1–5 years prior to diagnosis, respectively (Table 2, Fig. 1). Study-specific estimates yielded similar results (ESM Tables 3, 4).

**Table 2:** Pooled ORs and 95% CIs for the association between antibiotic exposure and LADA risk within different exposure windows prior to diagnosis

Antibiotic dispensations	Broad spectrum			Narrow spectrum			Any type		
	Cases/controls (*n*)	Model 1	Model 2	Cases/controls (*n*)	Model 1	Model 2	Cases/controls (*n*)	Model 1	Model 2
		OR_pooled_ (95% CI)	OR_pooled_ (95% CI)		OR_pooled_ (95% CI)	OR_pooled_ (95% CI)		OR_pooled_ (95% CI)	OR_pooled_ (95% CI)
0 to <1 year exposure window									
No vs any									
0	592/3905	1.00	1.00	554/3753	1.00	1.00	498/3391	1.00	1.00
≥1	87/531	1.13 (0.87, 1.46)	1.10 (0.84, 1.43)	125/683	1.33 (1.07, 1.66)	1.19 (0.94, 1.49)	181/1045	1.24 (1.02, 1.51)	1.15 (0.93, 1.41)
1–5 years exposure window									
No vs any									
0	379/2572	1.00	1.00	312/2120	1.00	1.00	246/1655	1.00	1.00
≥1	181/1263	1.06 (0.87, 1.30)	1.03 (0.84, 1.27)	248/1715	1.06 (0.88, 1.28)	1.00 (0.83, 1.22)	314/2180	1.05 (0.87, 1.27)	0.98 (0.80, 1.20)
Number									
0	379/2572	1.00	1.00	312/2120	1.00	1.00	246/1655	1.00	1.00
1–2	137/936	1.07 (0.86, 1.33)	1.03 (0.82, 1.30)	193/1355	1.01 (0.83, 1.23)	0.98 (0.80, 1.21)	208/1426	1.05 (0.85, 1.29)	0.99 (0.80, 1.24)
3–4	29/204	1.08 (0.70, 1.66)	1.12 (0.72, 1.76)	43/249	1.43 (0.99, 2.06)	1.15 (0.78, 1.71)	72/434	1.37 (1.00, 1.86)	1.30 (0.94, 1.81)
≥5	15/123	1.07 (0.60, 1.90)	0.93 (0.51, 1.70)	12/111	0.94 (0.50, 1.77)	0.81 (0.42, 1.57)	34/320	1.15 (0.78, 1.70)	1.01 (0.67, 1.52)
Per dispensation	560/3835	1.00 (0.94, 1.06)	0.99 (0.93, 1.06)	560/3835	1.02 (0.96, 1.09)	1.00 (0.93, 1.07)	560/3835	1.01 (0.97, 1.05)	1.00 (0.95, 1.04)
6–10 years exposure window									
No vs any									
0	142/1000	1.00	1.00	92/779	1.00	1.00	74/598	1.00	1.00
≥1	80/584	0.96 (0.70, 1.31)	0.90 (0.65, 1.26)	130/805	1.47 (1.09, 1.99)	1.39 (1.01, 1.91)	148/986	1.34 (0.98, 1.84)	1.33 (0.95, 1.85)
Number									
0	142/1000	1.00	1.00	92/779	1.00	1.00	74/598	1.00	1.00
1–2	58/426	0.92 (0.65, 1.30)	0.86 (0.59, 1.23)	105/604	1.50 (1.09, 2.07)	1.44 (1.03, 2.02)	95/588	1.40 (1.00, 1.98)	1.45 (1.01, 2.09)
3–4	14/96	1.15 (0.61, 2.18)	1.14 (0.58, 2.24)	18/132	1.55 (0.87, 2.77)	1.32 (0.72, 2.44)	33/225	1.30 (0.81, 2.08)	1.11 (0.68, 1.82)
≥5	8/62	1.52 (0.65, 3.54)	1.63 (0.68, 3.93)	7/69	1.24 (0.52, 2.95)	1.21 (0.49, 2.98)	20/173	1.16 (0.67, 2.02)	1.15 (0.64, 2.06)
Per dispensation	222/1584	1.03 (0.93, 1.14)	1.04 (0.93, 1.16)	222/1584	1.07 (0.97, 1.18)	1.06 (0.95, 1.17)	222/1584	1.04 (0.98, 1.10)	1.03 (0.96, 1.10)
0–10 years exposure window									
No vs any									
0	111/748	1.00	1.00	68/496	1.00	1.00	52/338	1.00	1.00
≥1	111/836	1.03 (0.76, 1.39)	0.96 (0.70, 1.32)	154/1088	1.12 (0.81, 1.554)	1.01 (0.72, 1.42)	170/1246	1.02 (0.72, 1.45)	0.98 (0.67, 1.43)
Number									
0	111/748	1.00	1.00	68/496	1.00	1.00	52/338	1.00	1.00
1–2	64/510	0.97 (0.68, 1.36)	0.84 (0.58, 1.22)	90/650	1.03 (0.73, 1.47)	0.99 (0.68, 1.43)	72/552	0.92 (0.62, 1.37)	0.93 (0.61, 1.41)
3–4	27/166	1.25 (0.77, 2.02)	1.29 (0.78, 2.13)	36/229	1.21 (0.77, 1.91)	1.08 (0.67, 1.76)	–	–	–
≥5	20/160	1.04 (0.60, 1.78)	1.04 (0.59, 1.86)	28/209	1.31 (0,79, 2.18)	1.06 (0.62, 1.82)	52/416	1.05 (0.67, 1.63)	0.98(0.61, 1.58)
Per dispensation	222/1584	1.00 (0.95, 1.05)	1.01 (0.95, 1.06)	222/1584	1.02 (0.96, 1.07)	1.01 (0.95, 1.07)	222/1584	1.00 (0.97, 1.04)	1.00 (0.97, 1.04)

For exposure windows from 1–5 years upwards, only individuals with complete follow-up in the registers are included in each exposure window. For the 0–10 years exposure window, there were 0 cases with 3–4 dispensations in HUNT so the estimates could not be pooled. The total number of cases and controls with 3–4 dispensations was 46/278

Model 1 adjusted for age and sex

Model 2 additionally adjusted for BMI, smoking status, physical activity level, educational level and family history of diabetes

In the 6–10 years exposure window, narrow-spectrum, but not broad-spectrum, antibiotics were associated with an increased risk of LADA, with an OR_pooled_ of 1.39 (95% CI 1.01, 1.91) for one or more vs no dispensations (Table 2, Fig. 1). In the study-specific analyses, the increased risk of LADA was seen in the Swedish data (OR 1.48, 95% CI 1.05, 2.09), but not in the Norwegian data (OR 0.95, 95% CI 0.41, 2.18]) (ESM Tables 3, 4). The corresponding OR_pooled_ for broad-spectrum antibiotics was 0.90 (95% CI 0.65, 1.26) (Table 2, Fig. 1). In the 6–10 years exposure window, the increased risk of LADA associated with narrow-spectrum antibiotics in the ESTRID study was seen at ≥15 consecutive days of exposure vs 0 days, with an OR of 1.64 (95% CI 1.12, 2.39), with an OR of 1.23 (95% CI 0.77, 1.96) for 1–14 consecutive days of exposure vs 0 days (ESM Table 3). Similarly, the OR was 1.57 (95% CI 1.04, 2.36) for ≥20 cumulative days of exposure vs 0 days, and 1.40 (95% CI 0.93, 2.11) for 1–19 cumulative days of exposure vs 0 days (ESM Table 3). When combining the exposure windows to a 0–10 years window, the increased risk associated with narrow-spectrum antibiotics was completely attenuated, and no other associations were evident (Table 2, ESM Tables 3, 4).

### Antibiotic exposure and type 2 diabetes

Exposure to any type of antibiotic within the year prior to diagnosis was associated with an increased risk of type 2 diabetes (OR_pooled_ 1.19, 95% CI 1.05, 1.36, for one or more vs no dispensations), and this association was also evident for broad- and narrow-spectrum antibiotics separately (Table 3, Fig. 1). There was no association between type 2 diabetes and antibiotic dispensations in the 1–5 years exposure window, except for an OR_pooled_ of 1.33 (95% CI 1.08, 1.65) for three to four dispensations vs no dispensations of any type of antibiotic (Table 3). Study-specific and pooled estimates were similar (ESM Tables 5, 6). Analyses of the ESTRID data did not show an association between type 2 diabetes and consecutive or cumulative antibiotic exposure (ESM Table 5). Furthermore, there was no association between type 2 diabetes and antibiotic exposure in the 6–10 years exposure window (Table 3, ESM Tables 5, 6). Although there were statistically significant associations with both broad- and narrow-spectrum antibiotics in the unadjusted models (model 1), these associations did not generally remain in the lifestyle-adjusted analyses when antibiotic dispensations occurring >1 year prior to diagnosis was the exposure of interest.

**Table 3:** Pooled ORs and 95% CIs for the association between antibiotic exposure and type 2 diabetes risk within different exposure windows prior to diagnosis

Antibiotic dispensations	Broad spectrum			Narrow spectrum			Any type		
	Cases/controls (*n*)	Model 1	Model 2	Cases/controls (*n*)	Model 1	Model 2	Cases/controls (*n*)	Model 1	Model 2
		OR_pooled_ (95% CI)	OR_pooled_ (95% CI)		OR_pooled_ (95% CI)	OR_pooled_ (95% CI)		OR_pooled_ (95% CI)	OR_pooled_ (95% CI)
0 to <1-year exposure window									
No vs any									
0	2825/3905	1.00	1.00	2694/3753	1.00	1.00	2377/3391	1.00	1.00
≥1	519/531	1.33 (1.16, 1.54)	1.21 (1.02, 1.43)	650/683	1.45 (1.27, 1.65)	1.23 (1.06, 1.42)	967/1045	1.37 (1.23, 1.54)	1.19 (1.05, 1.36)
1–5 years exposure window									
No vs any									
0	1711/2572	1.00	1.00	1451/2120	1.00	1.00	1095/1655	1.00	1.00
≥1	984/1263	1.26 (1.12, 1.41)	1.07 (0.94, 1.23)	1244/1715	1.13 (1.02, 1.27)	0.98 (0.87, 1.12)	1600/2180	1.18 (1.06, 1.32)	1.00 (0.88, 1.13)
Number									
0	1711/2572	1.00	1.00	1451/2120	1.00	1.00	1095/1655	1.00	1.00
1–2	735/936	1.28 (1.13, 1.45)	1.11 (0.95, 1.29)	951/1355	1.08 (0.96, 1.21)	0.98 (0.85, 1.13)	1008/1426	1.16 (1.03, 1.30)	1.03 (0.89, 1.19)
3–4	156/204	1.24 (0.98, 1.58)	1.04 (0.78, 1.39)	214/249	1.44 (1.16, 1.80)	1.06 (0.83, 1.36)	351/434	1.52 (1.27, 1.83)	1.33 (1.08, 1.65)
≥5	93/123	1.18 (0.86, 1.61)	0.91 (0.63, 1.31)	79/111	1.24 (0.88, 1.74)	0.88 (0.59, 1.32)	241/320	1.38 (1.12, 1.70)	0.99 (0.78, 1.26)
Per dispensation	2695/3835	1.03 (1.00, 1.06)	1.01 (0.97, 1.04)	2695/3835	1.06 (1.02, 1.11)	1.00 (0.96, 1.05)	2695/3835	1.04 (1.01, 1.06)	1.01 (0.98, 1.03)
6–10 years exposure window									
No vs any									
0	672/1000	1.00	1.00	522/779	1.00	1.00	391/598	1.00	1.00
≥1	411/584	1.16 (1.00, 1.36)	0.97 (0.81, 1.15)	561/805	1.17 (0.98, 1.38)	0.94 (0.77, 1.15)	692/986	1.17 (0.98, 1.40)	0.97 (0.79, 1.19)
Number									
0	672/1000	1.00	1.00	522/779	1.00	1.00	391/598	1.00	1.00
1–2	294/426	0.99 (0.82, 1.21)	0.82 (0.65, 1.03)	415/604	1.12 (0.93, 1.34)	0.98 (0.79, 1.21)	409/588	1.16 (0.95, 1.41)	1.06 (0.85, 1.34)
3–4	76/96	1.07 (0.75, 1.53)	0.89 (0.59, 1.34)	99/132	1.42 (1.03, 1.95)	0.81 (0.55, 1.18)	159/225	1.20 (0.92, 1.57)	0.80 (0.58, 1.09)
≥5	41/62	1.08 (0.68, 1.70)	0.92 (0.55, 1.54)	47/69	1.22 (0.80, 1.88)	0.89 (0.54, 1.46)	124/173	1.25 (0.93, 1.68)	0.92 (0.65, 1.29)
Per dispensation	1083/1584	1.01 (0.96, 1.06)	0.97 (0.91, 1.04)	1083/1584	1.06 (1.01, 1.12)	0.98 (0.92, 1.05)	1083/1584	1.03 (0.99, 1.06)	0.98 (0.94, 1.02)
0–10 years exposure window									
No vs any									
0	461/748	1.00	1.00	301/496	1.00	1.00	195/338	1.00	1.00
≥1	622/836	1.31 (1.10, 1.56)	1.07 (0.87, 1.31)	782/1088	1.30 (1.07, 1.57)	1.10 (0.89, 1.37)	888/1246	1.35 (1.09, 1.68)	1.16 (0.90, 1.49)
Number									
0	461/748	1.00	1.00	301/496	1.00	1.00	195/338	1.00	1.00
1–2	377/510	1.32 (1.08, 1.60)	1.09 (0.87, 1.37)	455/650	1.22 (0.99, 1.50)	1.15 (0.91, 1.46)	375/552	1.24 (0.98, 1.57)	1.19 (0.90, 1.57)
3–4	123/166	1.27 (0.95, 1.70)	1.15 (0.82, 1.61)	170/229	1.32 (1.01, 1.73)	1.05 (0.77, 1.44)	212/278	1.39 (1.06, 1.84)	1.16 (0.84, 1.60)
≥5	122/160	1.28 (0.95, 1.72)	0.90 (0.64, 1.26)	157/209	1.65(1.23, 2.20)	1.04 (0.75, 1.46)	301/416	1.55 (1.19, 2.01)	1.15 (0.85, 1.56)
Per dispensation	1083/1584	1.01 (0.98, 1.04)	0.98 (0.95, 1.01)	1083/1584	1.04 (1.01, 1.08)	0.99 (0.95, 1.02)	1083/1584	1.02 (1.00, 1.03)	0.98 (0.96, 1.01)

For exposure windows from 1–5 years upwards, only individuals with complete follow-up in the registers are included in each exposure window

Model 1 adjusted for age and sex

Model 2 additionally adjusted for BMI, smoking status, physical activity level, educational level and family history of diabetes

### Sensitivity analyses

In ESTRID, compared with adjustment for lifestyle factors only (model 2), further adjustment for comorbidities and healthcare use, using an hd-PS, attenuated the increased risk of LADA associated with one or more vs no dispensations of narrow-spectrum antibiotics in the 6–10 years exposure window (OR 1.43, 95% CI 0.94, 2.19) (ESM Table 7). However, longer duration of exposure to any type of antibiotic remained associated with an increased risk of LADA even after additional adjustment for comorbidity, with ORs of 1.66 (95% CI 1.04, 2.67) for ≥15 consecutive days of exposure vs 0 days, and 1.72 (95% CI 1.03, 2.87) for ≥20 cumulative days of exposure vs 0 days. Adjusting for hd-PS did not change the overall findings in type 2 diabetes (ESM Table 8).

Stratification by median GADA level (high vs low) did not reveal marked differences in the association between LADA and any type of antibiotic. For example, the OR_pooled_ for one or more vs 0 dispensations of any type of antibiotic was 0.97 (95% CI 0.74, 1.28) for GADA_low_ and 1.03 (95% CI 0.80, 1.34) for GADA_high_ in the 1–5 years exposure window. Similarly, there were no differences between GADA_high_ and GADA_low_ for the association between any type of antibiotic and LADA in the 6–10 years exposure window (ESM Table 9). Lastly, in the negative control analyses using dispensations of dermatological drugs, there was no apparent effect on the risk of LADA or type 2 diabetes, although some of the ORs approached significance, for example the OR for LADA associated with one or more vs 0 dispensations was 1.38 (95% CI 0.95, 2.02) in the 0–10 years exposure window (ESM Table 10).

## Discussion

This study investigated the association between antibiotic exposure and LADA, using data from two Scandinavian population-based cohorts with linkage to national prescribed drug registers, with comparative analyses for type 2 diabetes. We observed no increased risk of LADA with exposure to broad-spectrum antibiotics up to 10 years prior to diagnosis. The Swedish but not the Norwegian data pointed to an increased risk from exposure to narrow-spectrum antibiotics 6–10 years prior to diagnosis. We found little evidence of an increased risk of type 2 diabetes with prior exposure to antibiotics.

### Results in relation to previous findings

Two nationwide studies from Denmark and Sweden noted an increased risk of type 1 diabetes in children exposed to antibiotics during their first years of life [4, 5], which in the Swedish study was further strengthened by similar findings using a quasi-experimental sibling design. In contrast to the Danish study [4], we observed no association between exposure to broad-spectrum antibiotics and LADA, and this was consistent over the different exposure windows and the two study populations. However, in line with the Swedish study [5], which found an increased risk of type 1 diabetes in children exposed to narrow-spectrum antibiotics, we observed an increased risk of LADA with prior exposure to narrow-spectrum antibiotics in adulthood, which was driven by the Swedish data. The increased risk was restricted to exposure 6–10 years prior to diagnosis. This may reflect an aetiologically relevant period, as GADA typically present several years before diabetes diagnosis [24]. Antibiotics can impact the composition of the gut microbiota [25], which has been suggested to play a role in the development of type 1 diabetes [26]. Broad-spectrum antibiotics likely have a more profound effect on gut microbiota [27] than narrow-spectrum antibiotics, but we did not observe an increased risk of LADA with exposure to the former. It is conceivable that narrow-spectrum antibiotics impact the gut microbiota in specific ways that promote an autoimmune process. Our finding of an increased risk of LADA with prior exposure to narrow-spectrum antibiotics in the Swedish data must, however, be interpreted with caution, as it was not replicated in the Norwegian data. Moreover, and in contrast to some studies in type 2 diabetes [11, 12], there was no dose–response relationship between the number of prescriptions of narrow-spectrum antibiotics and LADA risk. Importantly, this is the first study on antibiotic exposure and LADA risk and confirmations are clearly warranted.

Antibiotic exposure may be a proxy for infection susceptibility. In children, there is evidence of an increased risk of type 1 diabetes associated with infections [28], which may be mediated by antibiotic exposure. However, we recently showed that the risk of LADA is unrelated to prior diagnoses of infectious disease [29].

Studies in type 2 diabetes have consistently demonstrated an increased risk following antibiotic exposure [10–13, 30], even when accounting for confounding by lifestyle factors [10, 12]. Metabolic changes driven by alterations in gut microbiota has been proposed as a potential mechanism [26]. We could not confirm these previous findings in our fully adjusted models, except for an increased risk of type 2 diabetes with exposure to broad-spectrum antibiotics in the year prior to diagnosis. Residual confounding, even in studies adjusting for several lifestyle factors, may explain some of the previous associations. We had the advantage of being able to adjust for a range of comorbidities, including healthcare use, using a validated [31] algorithm to calculate hd-PS.

It is also possible that earlier observations were partly driven by reverse causation since a diagnosis of type 2 diabetes typically is preceded by a period with elevated glucose levels that may last several years. This is associated with an increased susceptibility to infections [32], and thus a higher chance of being prescribed antibiotics. A lag time of 6 months between antibiotic exposure and diagnosis has previously been applied [11], which may be too narrow a time window to mitigate potential effects of reverse causation. Notably, we observed an increased risk of type 2 diabetes only in people exposed to broad-spectrum antibiotics <1 year before diagnosis/index date, and not in the earlier exposure windows.

We used dispensations of dermatological drugs as a negative control exposure. These were not associated with LADA or type 2 diabetes, which gives an indication that cases in general were not dispensed more prescription drugs.

### Strengths and limitations

The strengths of this study include the use of two cohorts with linkage to nationwide prescription registers, the detailed adjustment for potential confounders, and the comparative analyses of type 2 diabetes. The prescribed drug registers in Sweden and Norway include all prescription drugs dispensed at pharmacies, resulting in nearly complete coverage and essentially unbiased exposure data. In HUNT, we only had information about the year of diagnosis for cases, leading to a cruder estimation of the exposure windows. To reduce the risk of any potential association being caused by reverse causation during the year prior to diagnosis, exposures from index year +1 were considered in HUNT. The Swedish and Norwegian prescription registers were initiated in 2005 and 2004, respectively, which meant that samples that were fully covered in the longer exposure windows were smaller. These samples, however, did not differ from the total population on the covariates used in the adjusted model.

We used age and GADA levels for classification of LADA and type 2 diabetes, with the addition of C-peptide levels in ESTRID, which primarily distinguishes LADA from type 1 diabetes. C-peptide levels at diagnosis were missing in HUNT and therefore it is possible that some individuals classified as having LADA had adult-onset type 1 diabetes. However, the effect of such misclassification is likely to be negligible, as adult type 1 diabetes is much less common than LADA [33]. There may also be some LADA and type 1 diabetes cases within the type 2 diabetes group, as we distinguished cases only according to GADA levels. However, GADA is present among >90% of all cases of adult-onset autoimmune diabetes, whereas only a small proportion are solely positive for autoantibodies other than GADA [34]. Sex stratification was not performed because our study was not sufficiently powered for such analyses; thus, there may be sex-specific associations that we were unable to detect. Finally, our results were obtained in a primarily Nordic population and may not a priori be generalisable to other ethnicities.

To conclude, our research does not suggest that prior exposure to broad-spectrum antibiotics increases the risk of developing LADA or type 2 diabetes when the exposure is measured up to 10 years before diabetes diagnosis. An increased risk of LADA related to narrow-spectrum antibiotics was noted. Further studies are warranted to verify these results.

## Supplementary Information

Below is the link to the electronic supplementary material.

ESM (CRDOWNLOAD 1072 KB)

## Data Availability

The de-identified datasets generated and analysed in the current study are available from the corresponding author on reasonable request (ESTRID) and with permission of the HUNT study by applying to the HUNT study data access committee.

## Abbreviations

ANDIS: All New Diabetics in Scania
ATC: Anatomical Therapeutic Chemical
ESTRID: Epidemiological Study of Risk Factors for LADA and Type 2 Diabetes
hd-PS: High-dimensional propensity scores
HUNT: Trøndelag Health Study
LADA: Latent autoimmune diabetes in adults
