# Supplementary material for: Exposure to antibiotics and risk of latent autoimmune diabetes in adults and type 2 diabetes: results from a Swedish case–control study (ESTRID) and the Norwegian HUNT study
Source: Diabetologia. 2024 Oct 28;68(1):69–81. doi: 10.1007/s00125-024-06302-5 (PMC11663149; doi:10.1007/s00125-024-06302-5)
Supplement: Supplementary file 1 — ESM (CRDOWNLOAD 1072 KB) [file 125_2024_6302_MOESM1_ESM.crdownload]

Supplemental material

Exposure to antibiotics and the risk of latent autoimmune diabetes in adults and type 2 diabetes-results from a Swedish case-control study and the Norwegian HUNT study

Jessica Edstorp[1], Marios Rossides[1,2], Emma Ahlqvist[3], Lars Alfredsson[1,4], Johan Askling[5], Daniela Di Giuseppe[5], Valdemar Grill[6], Elin P Sorgjerd[7, 8], Tiinamaija Tuomi[3,9,10,11,12], Bjørn Olav Åsvold[8,13], Sofia Carlsson[1]

[1]Institute of Environmental Medicine, Karolinska Institutet, Stockholm
[2]Department of Respiratory Medicine and Allergy, Theme Inflammation and Ageing, Karolinska University Hospital, Stockholm
[3]Department of Clinical Sciences in Malmö, Lund University Diabetes Centre, Lund University, Malmö
[4]Center for Occupational and Environmental Medicine, Region Stockholm, Stockholm
[5]Clinical Epidemiology Division, Department of Medicine Solna, Karolinska Institutet, Stockholm
[6]Department of Clinical and Molecular Medicine, Norwegian University of Science and Technology, Trondheim
[7]HUNT Research Center, Department of Public Health and Nursing, NTNU, Norwegian University of Science and Technology, Levanger
[8]Department of Endocrinology, Clinic of Medicine, St Olavs Hospital, Trondheim
[9]Institute for Molecular Medicine Finland, Helsinki University, Helsinki
[10]Division of Endocrinology, Abdominal Center, Helsinki University Hospital, Helsinki
[11]Research Program for Diabetes and Obesity, University of Helsinki, Helsinki
[12]Folkhälsan Research Center, Helsinki
[13]HUNT Center for Molecular and Clinical Epidemiology, Department of Public Health and Nursing, NTNU, Norwegian University of Science and Technology, Trondheim

Table of Contents

ESM Methods 3

ESM Table 1. Anatomical Therapeutic Classification (ATC) codes for included antibiotics and the classification into broad- or narrow-spectrum antibiotics 5

ESM Table 2. Number of dispensations (% of total) of the most common types of antibiotics in ESTRID and HUNT 1-5 years prior to index date 6

ESM Table 3. Odds ratios and 95% confidence intervals for the association between antibiotic exposure and the risk of LADA within different exposure windows prior to diagnosis/matching (ESTRID) 7

ESM Table 4. Odds ratios and 95% confidence intervals for the association between antibiotic exposure and the risk of LADA within different exposure windows prior to diagnosis/matching (HUNT). 9

ESM Table 5. Odds ratios and 95% confidence intervals for the association between antibiotic exposure and the risk of type 2 diabetes within different exposure windows prior to diagnosis/matching (ESTRID) 10

ESM Table 6. Odds ratios and 95% confidence intervals for the association between antibiotic exposure and the risk of type 2 diabetes within different exposure windows prior to diagnosis/matching (HUNT). 12

ESM Table 7. Odds ratios and 95% confidence intervals for the association between antibiotic exposure and the risk of LADA within different exposure windows prior to diagnosis/matching, adjusted for comorbidity (ESTRID) 13

ESM Table 8. Odds ratios and 95% confidence intervals for the association between antibiotic exposure and the risk of type 2 diabetes within different exposure windows prior to diagnosis/matching, adjusted for comorbidity (ESTRID) .. 14

ESM Table 9. Odds ratios and 95% confidence intervals for the association between exposure to any spectrum antibiotic and the risk of LADA, within different exposure windows prior to diagnosis/matching and stratified by GAD autoantibody level. 15

ESM Table 10. Odds ratios and 95% confidence intervals for the association between exposure to dermatologicals and the risk of LADA or type 2 diabetes, within different exposure windows prior to diagnosis/matching. (ESTRID). ... 16

ESM Figure 1. Study design 17

ESM Figure 2. Directed acyclic graph (DAG). 18

ESM Figure 3. Distribution of GADA levels among LADA cases in ESTRID 18

ESM Figure 4. Distribution of GADA levels among LADA cases in HUNT 19

References.. 20

ESM Methods

Epidemiological Study of Risk Factors for LADA and type 2 diabetes (ESTRID)

ESTRID is a Swedish case-control study with incident cases of LADA and type 2 diabetes recruited through the All New Diabetics in Scania (ANDIS) register and biobank. The aim of ANDIS is to clinically and genetically characterize all incident cases of diabetes in the county of Scania, Sweden [1]. All cases of LADA in ANDIS who consent to further studies are invited to ESTRID together with a random sample of type 2 diabetes cases (1:4). In addition, ESTRID enrolls incidence-density sampled control participants from the general population of Scania, identified through the Swedish Population Register, and matched on participation time (1:6), meaning that controls are recruited at the time of diagnosis of the case. This approach implies that a control can later become a case (i.e. the control is at risk of developing diabetes at the time of diagnosis of the case). Incidence-density sampling has the advantage that it allows for ORs to be interpreted as incidence rate ratios [2].

Furthermore, to secure proper control status in ESTRID, the randomly selected controls from the general population should 1) not be included in the ANDIS study at the time of recruitment, and 2) have responded “No” to the question “Have you ever suffered from and sought medical attention for the following illnesses?”- Diabetes (Yes/No) in the ESTRID questionnaire. If a person who has been recruited as a control instead responds “Yes” to this question, the person is dropped as a control. Type of diabetes is not considered at this instance, and the person will be dropped regardless of type if the answer is “Yes”. A small proportion (3.5% of LADA cases) was recruited through All New Diabetics in Uppsala (ANDiU), a sister study to ANDIS in Uppsala County, Sweden.

The Trøndelag Health Study (HUNT)

The HUNT study has invited the entire population of the Nord-Trøndelag county in Norway to a health examination on four occasions between 1984 and 2019 (HUNT1 1984–1986, HUNT2 1995–1997, HUNT3 2006–2008, HUNT4 2017–2019). We performed a nested case-control study in HUNT4 and partly in HUNT3 (cases diagnosed ≥2006 with matched controls). This was dictated by national prescription data being available from 2004. Control subjects were selected using an incidence-density sampling method among those that, at follow-up, responded “No” to the question “Have you had or do you have diabetes?”. No consideration of type of diabetes was made at this instance, and individuals were dropped as potential controls regardless of diabetes type if they responded “Yes” to the above question. For each year of follow-up between 2006 and 2019, we formed a risk set comprising all incident cases of that year along with individuals who at the time were at risk of developing LADA or type 2 diabetes (i.e. diabetes free at the time of diagnosis of the case). From this risk set, we randomly, in a single draw, selected 25 sex-matched controls per incident LADA case. This approach means that controls can be included more than once but ensures that they are only included once in each stratum.

Calculation of a high-dimensional propensity score

To adjust for unmeasured confounding related to comorbidity in ESTRID, we calculated high-dimensional propensity scores (hd-PS) using the Pharmacoepi Toolbox, Macro Version 2.4.18 [3]. This was not done in HUNT since we did not have access to data from the Norwegian Patient Register. The hd-PS included all covariates from the main analyses as well as ethnicity (born in Sweden: yes/no) and the ICD code (R73) indicating elevated blood glucose. Variables that are strongly associated with the exposure but may not be associated with the outcome should be removed from the data input before estimation of hd-PS since they can bias the effect of the exposure if strong unmeasured confounding is present. Therefore, ICD codes indicating infection and ATC codes for antibiotics were removed.

The Pharmacoepi Toolbox provides an algorithm for estimation of hd-PS from a large number of variables across multiple sources. In short, the steps are [4]:

1) *Identify data dimensions*
   We included four dimensions restricted to 6-10 years prior to index date: 1) Inpatient visit data classified using International Classification of Disease codes (ICD; 10th revision) from the National Patient Register (NPR) – 3 digit granularity, 2) ICD-10-coded outpatient visits (NPR) – 3-digit granularity 3) ICD-10-P-coded primary care visits (Scania health-care register) – 3 digit granularity, and 4) dispensations of prescribed medications identified through ATC codes (National Prescribed Drug Register) – 7 digit granularity.
2) *Identify empirical candidate covariates*
   In each of the four dimensions, the codes were sorted by prevalence and the 200 most prevalent codes from each dimension were identified. The option to include an indicator variable to infer the intensity of health service utilization was used [5].
3) *Assessment of recurrence*
   Within-patient frequency was assessed by the recurrence of each code considering three levels (once, sporadic, or frequent), resulting in 2400 indicator variables to be considered for inclusion in the propensity score.
4) *Prioritize covariates*
   Covariates were prioritized across data dimensions by their potential to bias the exposure-outcome relation.
5) *Select covariates*
   Finally, the algorithm selected the top covariates (k=500) to be included in the estimation of separate hd-PS for each analysis.

The algorithm for calculating hd-PS is specifically developed for binary exposures and outcomes, but we also included categorical exposures in our analyses. To use the macro for calculating hd-PS in these analyses, we created unique hd-PS for each of the levels in the categorical variable. We then included all the hd-PS corresponding to the different levels of the exposure in the analysis. It should be noted that this approach has not been suggested by the original authors, and in some analyses there was multicollinearity between the scores for the different levels, resulting in unstable estimates.

**ESM Table 1. Anatomical Therapeutic Classification (ATC) codes for included antibiotics and the classification into broad- or narrow-spectrum antibiotics.**			
**ATC code**	**Type of antibiotic**	**Narrow spectrum**	**Broad spectrum**
**A07AA**	**Antibiotics**		
A07AA09	Vancomycin (oral)	x	
**J01**	**Antibacterials for systemic use**		
J01AA	Tetracyclines		x
J01CA	Penicillins with extended spectrum		x
J01CE	Beta-lactamase sensitive penicillin	x	
J01CF	Beta-lactamase resistant penicillin	x	
J01CG	Beta-lactamase inhibitors		x
J01CR	Combinations of penicillins incl. beta-lactamase inhibitors		x
J01DB	First-generation cephalosporins		x
J01DC	Second-generation cephalosporins		x
J01DD	Third-generation cephalosporins		x
J01DF	Monobactams	x	
J01EA	Trimethoprim and derivatives	x	
J01EB	Short-acting sulfonamides	x	
J01EE	Combinations of sulfonamides and trimethoprim, incl. derivatives		x
J01FA	Macrolides	x	
J01FF	Lincosamides	x	
J01GB	Aminoglycoside antibacterials		x
J01MA	Fluoroguinolones		x
J01XA	Glycopeptide antibacterials	x	
J01XB	Polymyxins		x
J01XC	Steroid antibacterials	x	
J01XE	Nitrofuran derivatives	x	
J01XX01	Methenamine		x
J01XX08	Linezolid		x
**J04A**	**Drugs for treatment of tuberculosis**		
J04AB	Antibiotics	x	
**P01A**	**Agents against amoebiasis and other protozoal diseases**		
P01AB01	Metronidazole	x	

ESM Table 2. Number of dispensations (% of total) of the most common types of antibiotics in ESTRID and HUNT 1-5 years prior to index date (diagnosis or matching).

	ESTRID				HUNT			
**Type of antibiotic (ATC)**	No. of dispensations (% of total)							
	LADA	Type 2 diabetes	Controls	Total	LADA	Type 2 diabetes	Controls	Total
**Phenoxymethylpenicillin (J01CE02)**	275 (29.0)	948 (25.0)	1012 (26.6)	2235 (26.2)	33 (24.6)	394 (21.8)	652 (22.6)	1079 (22.3)
**Doxycycline (J01AA02)**	155 (16.4)	624 (16.5)	565 (14.8)	1344 (15.7)	16 (11.9)	235 (13.0)	336 (11.6)	587 (12.2)
**Flucloxacillin (J01CF05)**	83 (8.8)	398 (10.5)	291 (7.6)	772 (9.0)	-	-	-	-
**Pivimecillinam (J01CA08)**	80 (8.4)	240 (6.3)	358 (9.4)	678 (7.9)	14 (10.5)	226 (12.5)	516 (17.9)	756 (15.7)
**Ciprofloxacin (J01MA02)**	53 (5.6)	298 (7.9)	258 (6.8)	609 (7.1)	5 (3.7)	80 (4.4)	84 (2.9)	169 (3.5)
**Amoxicillin (J01CA04)**	47 (5.0)	236 (6.2)	263 (6.9)	546 (6.4)	7 (5.2)	137 (7.6)	164 (5.7)	308 (6.4)
**Dicloxacillin (J01CF01)**	-	-	-	-	12 (9.0)	103 (5.7)	195 (6.8)	310 (6.4)
**Erythromycin (J01FA01)**	31 (3.3)	68 (1.8)	66 (1.7)	165 (1.9)	11 (8.2)	106 (5.9)	140 (4.9)	257 (5.3)

ESM Table 3. Odds ratios and 95% confidence intervals for the association between antibiotic exposure and the risk of **LADA** within different exposure windows prior to diagnosis/matching (ESTRID).

	**Broad spectrum**			**Narrow spectrum**			**Any type**		
	**Cases/ controls (n)**	Model 1 **OR (95% CI)**	Model 2 **OR (95% CI)**	**Cases/ controls (n)**	Model 1 **OR (95% CI)**	Model 2 **OR (95% CI)**	**Cases/ controls (n)**	Model 1 **OR (95% CI)**	Model 2 **OR (95% CI)**
**0–<1-year exposure window**									
**No vs. any dispensation**									
0	525/2119	1.00	1.00	495/2029	1.00	1.00	446/1846	1.00	1.00
≥1	72/267	1.10 (0.83, 1.46)	1.06 (0.79, 1.42)	102/357	1.21 (0.95, 1.55)	1.08 (0.84, 1.40)	151/540	1.18 (0.95, 1.46)	1.08 (0.86, 1.35)
**1–5-year exposure window**									
**No vs. any dispensation**									
0	340/1625	1.00	1.00	281/1343	1.00	1.00	224/1057	1.00	1.00
≥1	162/760	1.10 (0.89, 1.37)	1.10 (0.88, 1.37)	221/1042	1.07 (0.88, 1.31)	1.03 (0.83, 1.26)	278/1328	1.04 (0.85, 1.27)	0.99 (0.80, 1.23)
**Number of dispensations**									
0	340/1625	1.00	1.00	281/1343	1.00	1.00	224/1057	1.00	1.00
1–2	124/577	1.10 (0.87, 1.39)	1.08 (0.84, 1.37)	175/863	1.02 (0.83, 1.26)	1.00 (0.80, 1.25)	187/928	0.98 (0.79, 1.22)	0.93 (0.74, 1.17)
3–4	28/128	1.16 (0.74, 1.79)	1.21 (0.77, 1.93)	37/124	1.49 (1.00, 2.22)	1.24 (0.81, 1.91)	64/240	1.37 (0.99, 1.89)	1.33 (0.94, 1.86)
≥5	10/55	1.00 (0.50, 2.03)	1.06 (0.51, 2.18)	9/55	0.91 (0.43, 1.89)	0.84 (0.39, 1.82)	27/160	0.94 (0.60, 1.47)	0.87 (0.55, 1.40)
Per dispensation	502/2385	1.01 (0.94, 1.07)	1.01 (0.94, 1.08)	502/2385	1.03 (0.96, 1.11)	1.01 (0.94, 1.10)	502/2385	1.02 (0.97, 1.06)	1.01 (0.96, 1.06)
**Consecutive exposure (days)**									
0	340/1625	1.00	1.00	281/1343	1.00	1.00	224/1057	1.00	1.00
1–14	124/544	1.19 (0.94, 1.51)	1.19 (0.93, 1.52)	89/397	1.15 (0.88, 1.50)	1.14 (0.86, 1.51)	127/564	1.12 (0.88, 1.44)	1.12 (0.87, 1.46)
≥15	38/216	0.90 (0.62, 1.30)	0.87 (0.59, 1.29)	132/645	1.03 (0.82, 1.30)	0.96 (0.75, 1.23)	151/764	0.98 (0.78, 1.24)	0.90 (0.71, 1.15)
**Cumulative expsoure (days)**									
0	340/1625	1.00	1.00	281/1343	1.00	1.00	224/1057	1.00	1.00
1–19	100/430	1.21 (0.93, 1.56)	1.19 (0.91, 1.56)	126/611	1.03 (0.82, 1.31)	1.04 (0.81, 1.34)	125/607	1.01 (0.79, 1.29)	1.02 (0.79, 1.32)
≥20	62/330	0.97 (0.72, 1.32)	0.97 (0.70, 1.33)	95/431	1.13 (0.87, 1.47)	1.00 (0.76, 1.33)	153/721	1.07 (0.85, 1.35)	0.97 (0.76, 1.24)
Per week	502/2385	1.00 (0.98, 1.02)	1.00 (0.97, 1.02)	502/2385	1.01 (0.99, 1.04)	1.01 (0.98, 1.04)	502/2385	1.00 (0.99, 1.01)	1.00 (0.99, 1.01)
**6–10-year exposure window**									
**No vs. any dispensation**									
0	123/540	1.00	1.00	78/421	1.00	1.00	63/331	1.00	1.00
≥1	70/319	1.02 (0.73, 1.44)	0.98 (0.68, 1.40)	115/438	1.54 (1.11, 2.14)	1.48 (1.05, 2.09)	130/528	1.42 (1.01, 2.00)	1.45 (1.01, 2.08)
**Number of dispensations**									
0	123/540	1.00	1.00	78/421	1.00	1.00	63/331	1.00	1.00
1–2	49/246	0.91 (0.63, 1.34)	0.85 (0.57, 1.27)	96/343	1.61 (1.14, 2.26)	1.57 (1.09, 2.25)	86/329	1.49 (1.03, 2.15)	1.59 (1.07, 2.35)
3–4	13/43	1.36 (0.70, 2.67)	1.35 (0.67, 2.72)	16/63	1.69 (0.90, 3.14)	1.53 (0.80, 2.94)	29/120	1.38 (0.83, 2.29)	1.21 (0.71, 2.07)
≥5	8/30	1.52 (0.65, 3.54)	1.63 (0.68, 3.93)	3/32	0.57 (0.17, 1.94)	0.53 (0.15, 1.86)	15/79	1.17 (0.62, 2.21)	1.23 (0.64, 2.38)
Per dispensation	193/859	1.06 (0.95, 1.18)	1.07 (0.95, 1.19)	193/859	1.05 (0.94, 1.17)	1.04 (0.92, 1.17)	193/859	1.05 (0.98, 1.12)	1.04 (0.97, 1.12)
**Consecutive exposure (days)**									

0	123/540	1.00	1.00	78/421	1.00	1.00	63/331	1.00	1.00
1–14	43/240	0.84 (0.57, 1.25)	0.81 (0.53, 1.22)	36/162	1.27 (0.81, 1.99)	1.23 (0.77, 1.96)	39/214	1.02 (0.65, 1.59)	1.08 (0.67, 1.73)
≥15	27/79	1.53 (0.94, 2.51)	1.45 (0.86, 2.46)	79/276	1.70 (1.29, 2.44)	1.64 (1.12, 2.39)	91/314	1.70 (1.18, 2.45)	1.69 (1.14, 2.49)
**Cumulative exposure (days)**									
0	123/540	1.00	1.00	78/421	1.00	1.00	63/331	1.00	1.00
1–19	31/193	0.76 (0.49, 1.18)	0.72 (0.45, 1.14)	56/236	1.38 (0.93, 2.03)	1.40 (0.93, 2.11)	41/222	1.06 (0.69, 1.64)	1.13 (0.72, 1.80)
≥20	39/126	1.42 (0.92, 2.17)	1.37 (0.87, 2.16)	59/202	1.73 (1.18, 2.56)	1.57 (1.04, 2.36)	89/306	1.70 (1.17, 2.47)	1.67 (1.13, 2.47)
Per week	193/859	1.01 (0.98, 1.05)	1.01 (0.97, 1.05)	193/859	1.03 (0.98, 1.08)	1.03 (0.98, 1.08)	193/859	1.01 (0.99, 1.04)	1.01 (0.99, 1.04)
**0–10-year exposure window**									
**No vs. any dispensation**									
0	99/442	1.00	1.00	60/272	1.00	1.00	46/200	1.00	1.00
≥1	94/417	1.08 (0.78, 1.50)	1.03 (0.73, 1.46)	133/587	1.08 (0.76, 1.52)	1.02 (0.71, 1.47)	147/659	1.05 (0.72, 1.53)	1.04 (0.69, 1.55)
**Number of dispensations**									
0	99/442	1.00	1.00	60/272	1.00	1.00	46/200	1.00	1.00
1–2	55/264	1.01 (0.70, 1.47)	0.90 (0.61, 1.35)	79/378	0.99 (0.68, 1.44)	0.97 (0.65, 1.44)	63/320	0.92 (0.60, 1.41)	0.94 (0.60, 1.48)
3–4	22/78	1.26 (0.74, 2.17)	1.29 (0.74, 2.27)	33/126	1.23 (0.76, 2.00)	1.12 (0.67, 1.88)	46/154	1.38 (0.86, 2.21)	1.29 (0.78, 2.15)
≥5	17/75	1.16 (0.64, 2.09)	1.27 (0.68, 2.36)	21/83	1.25 (0.71, 2.22)	1.13 (0.62, 2.06)	38/185	0.99 (0.61, 1.63)	0.98 (0.58, 1.66)
Per dispensation	193/859	1.01 (0.96, 1.08)	1.02 (0.96, 1.08)	193/859	1.01 (0.95, 1.08)	1.01 (0.94, 1.08)	193/859	1.01 (0.97, 1.05)	1.01 (0.97, 1.05)
**Cumulative exposure (days)**									
0	99/442	1.00	1.00	60/272	1.00	1.00	46/200	1.00	1.00
1–19	42/196	1.05 (0.69, 1.58)	1.00 (0.65, 1.54)	42/247	0.83 (0.53, 1.28)	0.89 (0.56, 1.41)	26/191	0.64 (0.38, 1.09)	0.70 (0.40, 1.22)
20–49	29/132	1.01 (0.63, 1.62)	0.93 (0.56, 1.53)	61/240	1.17 (0.78, 1.76)	1.04 (0.67, 1.60)	61/246	1.18 (0.76, 1.82)	1.18 (0.74, 1.88)
≥50	23/89	1.29 (0.76, 2.18)	1.29 (0.74, 2.25)	30/100	1.46 (0.88, 2.41)	1.27 (0.74, 2.17)	60/222	1.27 (0.82, 1.98)	1.16 (0.73, 1.86)
Per week	193/859	1.00 (0.98, 1.01)	1.00 (0.99, 1.02)	193/859	1.01 (0.98, 1.03)	1.01 (0.98, 1.03)	193/859	1.00 (0.99, 1.01)	1.00 (0.99, 1.01)

Model 1 adjusted for age and sex.
Model 2 adjusted for age, sex, BMI, smoking, physical activity, educational level, and family history of diabetes.

ESM Table 4. Odds ratios and 95% confidence intervals for the association between antibiotic exposure and the risk of **LADA** within different exposure windows prior to matching (HUNT).

	Broad spectrum			Narrow spectrum			Any type		
	**Cases/ controls (n)**	Model 1 **OR (95% CI)**	Model 2 **OR (95% CI)**	**Cases/ controls (n)**	Model 1 **OR (95% CI)**	Model 2 **OR (95% CI)**	**Cases/ controls (n)**	Model 1 **OR (95% CI)**	Model 2 **OR (95% CI)**
**0–<1-year exposure window**									
**No vs. any dispensation**									
0	67/1786	1.00	1.00	59/1724	1.00	1.00	52/1545	1.00	1.00
≥1	15/264	1.26 (0.70, 2.27)	1.26 (0.69, 2.31)	23/326	1.98 (1.20, 3.27)	1.77 (1.04, 2.99)	30/505	1.60 (1.00, 2.55)	1.51 (0.93, 2.46)
**1–5-year exposure window**									
**No vs. any dispensation**									
0	39/947	1.00	1.00	31/777	1.00	1.00	22/598	1.00	1.00
≥1	19/503	0.81 (0.46, 1.44)	0.64 (0.35, 1.18)	27/673	1.00 (0.59, 1.71)	0.83 (0.47, 1.45)	36/852	1.12 (0.64, 1.94)	0.90 (0.51, 1.61)
**Number of dispensations**									
0	39/947	1.00	1.00	31/777	1.00	1.00	22/598	1.00	1.00
1–2	13/359	0.84 (0.44, 1.60)	0.71 (0.36, 1.40)	18/492	0.95 (0.52, 1.74)	0.86 (0.46, 1.60)	21/498	1.19 (0.64, 2.20)	1.06 (0.56, 2.01)
3–4	1/76	0.26 (0.04, 1.96)	0.24 (0.03, 1.88)	6/125	1.16 (0.47, 2.87)	0.78 (0.29, 2.07)	8/194	1.15 (0.50, 2.66)	0.91 (0.38, 2.19)
≥5	5/68	1.23 (0.45, 3.36)	0.68 (0.23, 2.06)	3/56	1.02 (0.29, 3.52)	0.73 (0.20, 2.68)	7/160	0.91 (0.37, 2.22)	0.54 (0.20, 1.45)
Per dispensation	58/1450	0.96 (0.84, 1.11)	0.89 (0.75, 1.06)	58/1450	0.99 (0.84, 1.17)	0.92 (0.76, 1.11)	58/1450	0.98 (0.90, 1.07)	0.94 (0.84, 1.04)
**6–10-year exposure window**									
**No vs. any dispensation**									
0	19/460	1.00	1.00	14/358	1.00	1.00	11/267	1.00	1.00
≥1	10/265	0.69 (0.31, 1.54)	0.58 (0.25, 1.37)	15/367	1.15 (0.53, 2.50)	0.95 (0.41, 2.18)	18/458	0.99 (0.45, 2.21)	0.80 (0.34, 1.89)
**Number of dispensations**									
0	19/460	1.00	1.00	14/358	1.00	1.00	11/267	1.00	1.00
1–2	9/180	0.98 (0.42, 2.28)	0.89 (0.36, 2.16)	9/261	0.96 (0.40, 2.32)	0.82 (0.32, 2.10)	9/259	0.96 (0.38, 2.44)	0.81 (0.30, 2.19)
3–4	1/53	0.27 (0.04, 2.16)	0.13 (0.01, 1.50)	2/69	0.91 (0.19, 4.26)	0.42 (0.07, 2.65)	4/105	0.92 (0.27, 3.08)	0.69 (0.19, 2.46)
≥5	0/32	-	-	4/37	2.77 (0.80, 9.62)	2.91 (0.79, 10.67)	5/94	1.13 (0.36, 3.51)	0.91 (0.26, 3.14)
Per dispensation	29/725	0.80 (0.59, 1.10)	0.75 (0.52, 1.09)	29/725	1.13 (0.93, 1.36)	1.11 (0.90, 1.37)	29/725	0.99 (0.86, 1.13)	0.97 (0.83, 1.13)
**0–10-year exposure window**									
**No vs. any dispensation**									
0	12/306	1.00	1.00	8/224	1.00	1.00	6/138	1.00	1.00
≥1	17/419	0.78 (0.36, 1.72)	0.63 (0.27, 1.48)	21/501	1.37 (0.58, 3.23)	0.96 (0.38, 2.39)	23/587	0.88 (0.34, 2.26)	0.69 (0.25, 1.88)
**Number of dispensations**									
0	12/306	1.00	1.00	8/224	1.00	1.00	6/138	1.00	1.00
1–2	9/246	0.73 (0.29, 1.82)	0.56(0.21, 1.53)	11/272	1.38 (0.53, 3.58)	1.09 (0.40, 2.97)	9/232	0.90 (0.30, 2.66	0.86 (0.27, 2.72)
3–4	5/88	1.19 (0.39, 3.65)	1.27 (0.40, 4.01)	3/103	1.05 (0.27, 4.14)	0.83 (0.20, 3.45)	0/124	-	-
≥5	3/85	0.58 (0.15, 2.24)	0.32 (0.07, 1.48)	7/126	1.58 (0.53, 4.70)	0.81 (0.24, 2.79)	14/231	1.35(0.48, 3.78)	0.98 (0.32, 2.99)
Per dispensation	29/725	0.94 (0.82, 1.07)	0.90 (0.76, 1.06)	29/725	1.03 (0.93, 1.15)	0.99 (0.87, 1.13)	29/725	0.99 (0.93, 1.05)	0.97 (0.89, 1.05)

Model 1 adjusted for age and sex.
Model 2 adjusted for age, sex, BMI, smoking, physical activity, educational level, and family history of diabetes.

ESM Table 5. Odds ratios and 95% confidence intervals for the association between antibiotic exposure and the risk of **type 2 diabetes** within different exposure windows prior to matching (ESTRID).

	**Broad spectrum**			**Narrow spectrum**			**Any type**		
	**Cases/ controls (n)**	Model 1 **OR (95% CI)**	Model 2 **OR (95% CI)**	**Cases/ controls (n)**	Model 1 **OR (95% CI)**	Model 2 **OR (95% CI)**	**Cases/ controls (n)**	Model 1 **OR (95% CI)**	Model 2 **OR (95% CI)**
**0-<1-year exposure window**									
**No vs. any dispensation**									
0	1763/2119	1.00	1.00	1680/2029	1.00	1.00	1498/1846	1.00	1.00
≥1	302/267	1.38 (1.15, 1.65)	1.26 (1.01, 1.58)	385/357	1.43 (1.21, 1.68)	1.19 (0.98, 1.45)	567/540	1.37 (1.19, 1.58)	1.17 (0.99, 1.40)
**1–5-year exposure window**									
**No vs. any dispensation**									
0	1224/1625	1.00	1.00	1050/1343	1.00	1.00	797/1057	1.00	1.00
≥1	693/760	1.28 (1.12, 1.47)	1.10 (0.94, 1.30)	867/1042	1.14 (1.00, 1.30)	1.01 (0.87, 1.18)	1120/1328	1.17 (1.03, 1.33)	1.00 (0.86, 1.17)
**Number of dispensations**									
0	1224/1625	1.00	1.00	1050/1343	1.00	1.00	797/1057	1.00	1.00
1–2	532/577	1.30 (1.12, 1.50)	1.12 (0.93, 1.34)	680/863	1.07 (0.93, 1.23)	1.00 (0.84, 1.17)	736/928	1.08 (0.94, 1.24)	0.94 (0.80, 1.12)
3–4	108/128	1.18 (0.89, 1.57)	0.98 (0.69, 1.39)	141/124	1.59 (1.21, 2.09)	1.12 (0.81, 1.55)	237/240	1.41 (1.13, 1.75)	1.20 (0.92, 1.55)
≥5	53/55	1.40 (0.92, 2.12)	1.26 (0.77, 2.04)	46/55	1.32 (0.85, 2.04)	1.01 (0.59, 1.71)	147/160	1.42 (1.09, 1.85)	1.07 (0.78, 1.47)
Per dispensation	1917/2385	1.05 (1.01, 1.10)	1.03 (0.98, 1.08)	1917/2385	1.08 (1.03, 1.14)	1.02 (0.97, 1.09)	1917/2385	1.05 (1.02, 1.08)	1.02 (0.99, 1.06)
**Consecutive exposure (days)**									
0	1224/1625	1.00	1.00	1050/1343	1.00	1.00	797/1057	1.00	1.00
1–14	473/544	1.25 (1.07, 1.46)	1.08 (0.90, 1.31)	303/397	1.15 (0.88, 1.50)	0.94 (0.76, 1.17)	430/564	1.07 (0.91, 1.26)	1.12 (0.86, 1.46)
≥15	220/216	1.37 (1.11, 1.70)	1.16 (0.89, 1.50)	564/645	1.03 (0.82, 1.30)	1.05 (0.88, 1.26)	690/764	1.24 (1.07, 1.44)	0.90 (0.71, 1.15)
**Cumulative exposure (days)**									
0	1224/1625	1.00	1.00	1050/1343	1.00	1.00	797/1057	1.00	1.00
1–19	397/430	1.32 (1.12, 1.56)	1.13 (0.92, 1.38)	458/611	1.01 (0.87, 1.18)	0.96 (0.79, 1.15)	449/607	1.00 (0.85, 1.18)	0.93 (0.77, 1.14)
≥20	296/330	1.24 (1.03, 1.49)	1.07 (0.86, 1.34)	409/431	1.33 (1.12, 1.57)	1.08 (0.89, 1.33)	671/721	1.32 (1.13, 1.53)	1.06 (0.88, 1.27)
Per week	1917/2385	1.00 (0.99, 1.02)	1.00 (0.99, 1.01)	1917/2385	1.03 (1.01, 1.05)	1.02 (0.99, 1.04)	1917/2385	1.01 (1.00, 1.02)	1.00 (0.99, 1.01)
**6–10-year exposure window**									
**No vs. any dispensation**									
0	444/540	1.00	1.00	348/421	1.00	1.00	257/331	1.00	1.00
≥1	283/319	1.12 (0.90, 1.38)	0.92 (0.71, 1.18)	379/438	1.15 (0.94, 1.42)	0.91 (0.71, 1.17)	470/528	1.24 (1.00, 1.54)	1.03 (0.80, 1.34)
**Number of dispensations**									
0	444/540	1.00	1.00	348/421	1.00	1.00	257/331	1.00	1.00
1–2	214/246	1.10 (0.87, 1.39)	0.89 (0.68, 1.18)	285/343	1.09 (0.87, 1.35)	0.93 (0.71, 1.21)	286/329	1.20 (0.94, 1.51)	1.12 (0.85, 1.48)
3–4	51/43	1.35 (0.86, 2.10)	1.11 (0.65, 1.89)	66/63	1.53 (1.03, 2.27)	0.85 (0.53, 1.38)	112/120	1.37 (0.99, 1.89)	0.87 (0.59, 1.28)
≥5	18/30	0.84 (0.45, 1.58)	0.79 (0.39, 1.63)	28/32	1.20 (0.69, 2.07)	0.88 (0.46, 1.69)	72/79	1.29 (0.88, 1.88)	0.95 (0.60, 1.48)
Per dispensation	727/859	1.02 (0.94, 1.09)	0.99 (0.90, 1.08)	727/859	1.06 (0.98, 1.13)	0.97 (0.89, 1.06)	727/859	1.03 (0.98, 1.08)	0.98 (0.93, 1.04)
**Consecutive exposure (days)**									
0	444/540	1.00	1.00	348/421	1.00	1.00	257/331	1.00	1.00
1–14	201/240	1.07 (0.84, 1.35)	0.87 (0.66, 1.16)	114/162	0.91 (0.68, 1.21)	0.68 (0.48, 0.96)	162/214	1.01 (0.77, 1.33)	0.85 (0.61, 1.18)

≥15	82/79	1.26 (0.89, 1.79)	1.03 (0.69, 1.56)	265/276	1.30 (1.03, 1.64)	1.07 (0.81, 1.41)	308/314	1.41 (1.11, 1.79)	1.17 (0.88, 1.55)
**Cumulative exposure (days)**[a]									
0	444/540	1.00	1.00	348/421	1.00	1.00	257/331	1.00	1.00
1–19	155/193	1.05 (0.81, 1.36)	0.84 (0.61, 1.14)	177/236	0.96 (0.74, 1.23)	0.86 (0.64, 1.16)	178/222	1.10 (0.85, 1.44)	1.01 (0.74, 1.39)
≥20	128/126	1.21 (0.91, 1.63)	1.04 (0.73, 1.46)	202/202	1.40 (1.09, 1.81)	0.97 (0.72, 1.32)	292/306	1.35 (1.06, 1.72)	1.05 (0.79, 1.40)
Per week	727/859	1.00 (0.97, 1.02)	0.99 (0.96, 1.02)	727/859	1.04 (1.00, 1.07)	1.01 (0.98, 1.05)	727/859	1.01 (0.99, 1.03)	1.00 (0.98, 1.02)
**0–10-year exposure window**									
**No vs. any dispensation**									
0	309/442	1.00	1.00	216/272	1.00	1.00	142/200	1.00	1.00
≥1	418/417	1.50 (1.22, 1.85)	1.20 (0.93, 1.53)	511/587	1.17 (0.93, 1.46)	1.03 (0.79, 1.34)	585/659	1.30 (1.01 1.67)	1.12 (0.83, 1.52)
**Number of dispensations**									
0	309/442	1.00	1.00	216/272	1.00	1.00	142/200	1.00	1.00
1–2	266/264	1.53 (1.21, 1.94)	1.22 (0.93, 1.61)	300/378	1.05 (0.82, 1.34)	1.03 (0.78, 1.38)	258/320	1.17 (0.89, 1.55)	1.14 (0.82, 1.59)
3–4	83/78	1.50 (1.05, 2.14)	1.32 (0.86, 2.02)	113/126	1.21 (0.87, 1.67)	0.93 (0.63, 1.37)	139/154	1.28 (0.92, 1.77)	1.01 (0.69, 1.49)
≥5	69/75	1.39 (0.95, 2.03)	0.97 (0.62, 1.52)	98/83	1.74 (1.21, 2.50)	1.18 (0.77, 1.82)	188/185	1.59 (1.16, 2.18)	1.19 (0.82, 1.74)
Per dispensation	727/859	1.02 (0.98, 1.06)	0.99 (0.94, 1.03)	727/859	1.05 (1.01, 1.10)	1.00 (0.95, 1.06)	727/859	1.02 (1.00, 1.05)	0.99 (0.96, 1.03)
**Cumulative exposure (days)**									
0	309/442	1.00	1.00	216/272	1.00	1.00	142/200	1.00	1.00
1–19	189/196	1.52 (1.17, 1.97)	1.21 (0.89, 1.65)	187/247	1.01 (0.77, 1.33)	1.10 (0.80, 1.52)	146/191	1.10 (0.80, 1.50)	1.13 (0.78, 1.64)
20–49	146/132	1.59 (1.19, 2.12)	1.31 (0.93, 1.84)	188/240	1.04 (0.79, 1.36)	0.82 (0.59, 1.13)	211/246	1.27 (0.95, 1.71)	1.09 (0.77, 1.55)
≥50	83/89	1.33 (0.94, 1.89)	0.98 (0.65, 1.49)	136/100	1.93 (1.39, 2.68)	1.40 (0.95, 2.06)	228/222	1.53 (1.13, 2.05)	1.15 (0.81, 1.64)
Per week	727/859	1.00 (0.99, 1.01)	0.99 (0.98, 1.01)	727/859	1.02 (1.00, 1.04)	1.01 (0.99, 1.03)	727/859	1.00 (0.99, 1.01)	1.00 (0.99, 1.01)

Model 1 adjusted for age and sex.

Model 2 adjusted for age, sex, BMI, smoking, physical activity, educational level, and family history of diabetes.

ESM Table 6. Odds ratios and 95% confidence intervals for the association between antibiotic exposure and the risk of **T2D** within different exposure windows prior to matching (HUNT).

	Broad spectrum			Narrow spectrum			Any type		
	**Cases/ controls (n)**	Model 1 **OR (95% CI)**	Model 2 **OR (95% CI)**	**Cases/ controls (n)**	Model 1 **OR (95% CI)**	Model 2 **OR (95% CI)**	**Cases/ controls (n)**	Model 1 **OR (95% CI)**	Model 2 **OR (95% CI)**
**0–<1-year exposure window**									
**No vs. any dispensation**									
0	1062/1786	1.00	1.00	1014/1724	1.00	1.00	879/1545	1.00	1.00
≥1	217/264	1.26 (1.00, 1.58)	1.15 (0.89, 1.48)	265/326	1.48 (1.20, 1.83)	1.28 (1.02, 1.62)	400/505	1.38 (1.15, 1.66)	1.22 (1.00, 1.49)
**1–5-year exposure window**									
**No vs. any dispensation**									
0	487/947	1.00	1.00	401/777	1.00	1.00	298/598	1.00	1.00
≥1	291/503	1.21 (0.97, 1.50)	1.01 (0.79, 1.29)	377/673	1.12 (0.91, 1.38)	0.93 (0.74, 1.17)	480/852	1.21 (0.98, 1.49)	0.99 (0.79, 1.26)
**Number of dispensations**									
0	487/947	1.00	1.00	401/777	1.00	1.00	298/598	1.00	1.00
1–2	203/359	1.23 (0.96, 1.57)	1.08 (0.82, 1.42)	271/492	1.10 (0.88, 1.38)	0.94 (0.73, 1.21)	272/498	1.42 (1.12, 1.80)	1.29 (0.99, 1.68)
3–4	48/76	1.42 (0.90, 2.24)	1.18 (0.71, 1.95)	73/125	1.21 (0.84, 1.75)	0.98 (0.66, 1.45)	114/194	1.83 (1.30, 2.56)	1.66 (1.14, 2.41)
≥5	40/68	0.94 (0.58, 1.51)	0.61 (0.35, 1.04)	33/56	1.12 (0.65, 1.93)	0.74 (0.40, 1.35)	94/160	1.32 (0.94, 1.85)	0.89 (0.61, 1.30)
Per dispensation	778/1450	1.02 (0.98, 1.06)	0.98 (0.93, 1.03)	778/1450	1.04 (0.98, 1.11)	0.98 (0.91, 1.05)	778/1450	1.02 (0.99, 1.05)	0.99 (0.95, 1.02)
**6–10-year exposure window**									
**No vs. any dispensation**									
0	228/460	1.00	1.00	174/358	1.00	1.00	134/267	1.00	1.00
≥1	128/265	0.84 (0.61, 1.15)	0.73 (0.51, 1.04)	182/367	1.20 (0.89, 1.62)	0.99 (0.70, 1.39)	222/458	1.05 (0.77, 1.43)	0.87 (0.62, 1.24)
**Number of dispensations**									
0	228/460	1.00	1.00	174/358	1.00	1.00	134/267	1.00	1.00
1–2	80/180	0.78 (0.54, 1.12)	0.69 (0.46, 1.04)	130/261	1.18 (0.85, 1.64)	1.07 (0.74, 1.54)	123/259	1.06 (0.74, 1.51)	0.96 (0.65, 1.43)
3–4	25/53	0.73 (0.41, 1.30)	0.66 (0.35, 1.23)	33/69	1.23 (0.72, 2.10)	0.74 (0.40, 1.38)	47/105	0.91 (0.57, 1.46)	0.67 (0.39, 1.14)
≥5	23/32	1.42 (0.73, 2.75)	1.08 (0.51, 2.27)	19/37	1.26 (0.63, 2.52)	0.90 (0.42,1.93)	52/94	1.19 (0.74, 1.90)	0.88 (0.52, 1.48)
Per dispensation	356/725	1.00 (0.93, 1.07)	0.96 (0.88, 1.05)	356/725	1.07 (0.98, 1.17)	1.00 (0.90, 1.10)	356/725	1.02 (0.97, 1.07)	0.98 (0.93, 1.04)
**0–10-year exposure window**									
**No vs. any dispensation**									
0	152/306	1.00	1.00	85/224	1.00	1.00	53/138	1.00	1.00
≥1	204/419	0.96 (0.70, 1.32)	0.85 (0.60, 1.21)	271/501	1.66 (1.17, 2.35)	1.28 (0.87, 1.88)	303/587	1.51 (1.00, 2.28)	1.26 (0.80, 2.00)
**Number of dispensations**									
0	152/306	1.00	1.00	85/224	1.00	1.00	53/138	1.00	1.00
1–2	111/246	0.93 (0.65, 1.33)	0.85 (0.57, 1.27)	155/272	1.76 (1.20, 2.57)	1.48 (0.97, 2.27)	117/232	1.45 (0.91, 2.29)	1.31(0.79, 2.20)
3–4	40/88	0.89 (0.53, 1.49)	0.91 (0.52, 1.58)	57/103	1.61 (0.99, 2.63)	1.33 (0.78, 2.29)	73/124	1.73 (1.03, 2.92)	1.58 (0.89, 2.82)
≥5	53/85	1.12 (0.70, 1.80)	0.80 (0.47, 1.37)	59/126	1.50 (0.93, 2.40)	0.86 (0.50, 1.47)	113/231	1.46 (0.92, 2.32)	1.07 (0.63, 1.79)
Per dispensation	356/725	1.00 (0.97, 1.04)	0.97 (0.93, 1.02)	356/725	1.03 (0.98, 1.08)	0.97 (0.92, 1.03)	356/725	1.01 (0.98, 1.03)	0.98 (0.95, 1.01)

Model 1 adjusted for age and sex.
Model 2 adjusted for age, sex, BMI, smoking, physical activity, educational level, and family history of diabetes.

ESM Table 7. Odds ratios and 95% confidence intervals for the association between antibiotic exposure and the risk of **LADA** within different exposure windows prior to diagnosis/matching, adjusted for comorbidity (ESTRID).

	**Broad spectrum**		**Narrow spectrum**		**Any antibiotic**	
	**Cases/ controls (n)**	**OR (95% CI)**	**Cases/ controls (n)**	**OR (95% CI)**	**Cases/ controls (n)**	**OR (95% CI)**
**1–5-year exposure window**						
**No vs. any dispensation**						
0	340/1625	1.00	281/1343	1.00	224/1057	1.00
≥1	162/760	1.01 (0.79, 1.28)	221/1042	1.05 (0.85, 1.31)	278/1328	0.91 (0.73, 1.14)
**Number of dispensations**						
0	340/1625	1.00	281/1343	1.00	224/1057	1.00
1–2	124/577	0.99 (0.77, 1.28)	175/863	0.98 (0.78, 1.23)	187/928	0.94 (0.74, 1.18)
3–4	28/128	1.32 (0.80, 2.17)	37/124	1.18 (0.74, 1.87)	64/240	1.16 (0.80, 1.69)
≥5	10/55	5.05 (0.47, 53.89)	9/55	1.63 (0.22, 12.10)	27/160	1.07 (0.65, 1.76)
**Consecutive duration of exposure (days)**						
0	340/1625	1.00	281/1343	1.00	224/1057	1.00
1-14	124/544	1.13 (0.88, 1.47)	89/397	1.10 (0.84, 1.44)	127/564	1.10 (0.84, 1.44)
≥15	38/216	0.74 (0.49, 1.14)	132/645	1.00 (0.79, 1.26)	151/764	0.93 (0.73, 1.20)
**Cumulative exposure (days)**						
0	340/1625	1.00	281/1343	1.00	224/1057	1.00
1–19	100/430	1.10 (0.83, 1.46)	126/611	0.98 (0.76, 1.26)	125/607	0.91 (0.70, 1.19)
≥20	62/330	0.88 (0.62, 1.24)	95/431	0.98 (0.73, 1.31)	153/721	0.91 (0.70, 1.18)
**6–10-year exposure window**						
**No vs. any dispensation**						
0	123/540	1.00	78/421	1.00	63/331	1.00
≥1	70/319	0.91 (0.54, 1.51)	115/438	1.43 (0.94, 2.19)	130/528	1.26 (0.79, 2.03)
**Number of dispensations**						
0	123/540	1.00	78/421	1.00	63/331	1.00
1–2	49/246	0.94 (0.57, 1.54)	96/343	1.48 (0.97, 2.24)	86/329	1.52 (0.99, 2.33)
3–4	13/43	0.33 (0.02, 5.65)	16/63	-	29/120	0.79 (0.25, 2.51)
≥5	8/30	1.95 (0.29, 13.07)	3/32	2.96 (0.06, 155.21)	15/79	2.87 (0.33, 24.74)
**Consecutive duration of exposure (days)**						
0	123/540	1.00	78/421	1.00	63/331	1.00
1-14	43/240	0.79 (0.46, 1.35)	36/162	1.24 (0.65, 2.35)	39/214	0.93 (0.52, 1.65)
≥15	27/79	1.74 (0.78, 3.90)	79/276	1.46 (0.92, 2.32)	91/314	1.66 (1.04, 2.67)
**Cumulative exposure (days)**						
0	123/540	1.00	78/421	1.00	63/331	1.00
1–19	31/193	0.72 (0.41, 1.28)	56/236	1.63 (0.99, 2.66)	41/222	1.22 (0.73, 2.05)
≥20	39/126	2.07 (0.89, 4.81)	59/202	1.49 (0.85, 2.59)	89/306	1.72 (1.03, 2.87)

Models adjusted for age, sex, and ethnicity, in addition to a high-dimensional propensity score estimated based on BMI, smoking, physical activity, educational level, family history of diabetes, elevated blood glucose, and 500 selected covariates from Patient and Prescription registers including an indicator for healthcare utilization.

ESM Table 8. Odds ratios and 95% confidence intervals for the association between antibiotic exposure and the risk of **type 2 diabetes** within different exposure windows prior to diagnosis/matching, adjusted for comorbidity (ESTRID).

	**Broad spectrum**		**Narrow spectrum**		**Any antibiotic**	
	**Cases/ controls (n)**	**OR (95% CI)**	**Cases/ controls (n)**	**OR (95% CI)**	**Cases/ controls (n)**	**OR (95% CI)**
**1–5-year exposure window**						
**No vs. any dispensation**						
0	1224/1625	1.00	1050/1343	1.00	797/1057	1.00
≥1	693/760	1.07 (0.92, 1.24)	867/1042	0.96 (0.83, 1.10)	1120/1328	0.97 (0.84, 1.11)
**Number of dispensations**						
0	1224/1625	1.00	1050/1343	1.00	797/1057	1.00
1–2	532/577	1.08 (0.92, 1.27)	680/863	0.99 (0.85, 1.14)	736/928	0.98 (0.84, 1.13)
3–4	108/128	1.08 (0.77, 1.50)	141/124	1.27 (0.94, 1.72)	237/240	1.15 (0.90, 1.46)
≥5	53/55	0.80 (0.07, 9.07)	46/55	0.35 (0.06, 1.97)	147/160	1.39 (1.03, 1.87)
**Consecutive duration of exposure (days)**						
0	1224/1625	1.00	1050/1343	1.00	797/1057	1.00
1-14	473/544	1.03 (0.87, 1.22)	303/397	0.95 (0.78, 1.15)	430/564	0.94 (0.78, 1.12)
≥15	220/216	1.07 (0.84, 1.37)	564/645	0.95 (0.81, 1.12)	690/764	0.98 (0.84, 1.15)
**Cumulative exposure (days)**						
0	1224/1625	1.00	1050/1343	1.00	797/1057	1.00
1–19	397/430	1.08 (0.90, 1.30)	458/611	0.94 (0.80, 1.11)	449/607	0.93 (0.78, 1.11)
≥20	296/330	1.02 (0.82, 1.26)	409/431	1.03 (0.85, 1.24)	671/721	1.00 (0.85, 1.18)
**6–10-year exposure window**						
**No vs. any dispensation**						
0	283/319	1.00	348/421	1.00	257/331	1.00
≥1	444/540	0.92 (0.67, 1.28)	379/438	1.06 (0.80, 1.40)	470/528	1.04 (0.76, 1.42)
**Number of dispensations**						
0	444/540	1.00	348/421	1.00	257/331	1.00
1–2	214/246	0.92 (0.66, 1.28)	285/343	1.11 (0.85, 1.46)	286/329	1.25 (0.95, 1.66)
3–4	51/43	0.84 (0.14, 4.94)	66/63	0.56 (0.01, 26.28)	112/120	1.23 (0.60, 2.54)
≥5	18/30	1.41 (0.46, 4.37)	28/32	8.03 (0.63, 102.28)	72/79	1.36 (0.42, 4.45)
**Consecutive duration of exposure (days)**						
0	444/540	1.00	348/421	1.00	257/331	1.00
1-14	201/240	0.93 (0.66, 1.32)	114/162	0.82 (0.54, 1.27)	162/214	0.94 (0.65, 1.36)
≥15	82/79	0.83 (0.46,1.51)	265/276	1.19 (0.87, 1.62)	308/314	1.24 (0.90, 1.70)
**Cumulative exposure (days)**						
0	444/540	1.00	348/421	1.00	257/331	1.00
1–19	155/193	1.06 (0.74, 1.51)	177/236	1.20 (0.86, 1.68)	178/222	1.26 (0.90, 1.75)
≥20	128/126	0.95 (0.50, 1.83)	202/202	1.16 (0.79, 1.70)	292/306	1.12 (0.79, 1.58)

Models adjusted for age, sex, and ethnicity, in addition to a high-dimensional propensity score estimated based on BMI, smoking, physical activity, educational level, family history of diabetes, elevated blood glucose, and 500 selected covariates from Patient and Prescription registers including an indicator for healthcare utilization.

ESM Table 9. Odds ratios and 95% confidence intervals for the association between exposure to **any type of antibiotic and the risk of LADA**, within different exposure windows prior to diagnosis/matching and stratified by GAD autoantibody level.

	**ESTRID**				**HUNT**				**POOLED**			
	**$GADA_{low}$**		**$GADA_{high}$**		**$GADA_{low}$**		**$GADA_{high}$**		**$GADA_{low}$**		**$GADA_{high}$**	
	**Cases/ controls (n)**	**OR (95% CI)**	**Cases/ controls (n)**	**OR (95% CI)**	**Cases/ controls (n)**	**OR (95% CI)**	**Cases/ controls (n)**	**OR (95% CI)**	**Cases/ controls (n)**	**OR (95% CI)**	**Cases/ controls (n)**	**OR (95% CI)**
**0–<1-year exposure window**												
**No vs. any dispensation**												
0	216/1846	1.00	220/1846	1.00	24/1545	1.00	25/1545	1.00	240/3391	1.00	245/3391	1.00
≥1	69/540	1.00 (0.73, 1.37)	81/540	1.19 (0.89, 1.59)	14/505	1.65 (0.81, 3.38)	13/505	1.27 (0.63, 2.57)	83/1045	1.08 (0.81, 1.45)	94/1045	1.20 (0.92,1.57)
**1–5-year exposure window**												
**No vs. any dispensation**												
0	103/1057	1.00	114/1057	1.00	10/598	1.00	12/598	1.00	113/1655	1.00	126/1655	1.00
≥1	128/1328	0.95 (0.71, 1.27)	146/1328	1.08 (0.82, 1.42)	19/852	1.18 (0.52, 2.68)	17/852	0.70 (0.32, 1.56)	147/2180	0.97 (0.74 1.28)	163/2180	1.03 (0.80, 1.34)
**Number of dispensations**												
0	103/1057	1.00	114/1057	1.00	10/598	1.00	12/598	1.00	113/1655	1.00	126/1655	1.00
1–2	89/928	0.90 (0.65, 1.24)	97/928	1.01 (0.75, 1.36)	10/498	1.17 (0.47, 2.94)	11/498	0.99 (0.42, 2.35)	99/1426	0.93 (0.68, 1.26)	108/1426	1.01 (0.76, 1.34)
3–4	31/240	1.41 (0.89, 2.23)	31/240	1.29 (0.83, 2.02)	6/194	2.02 (0.68, 5.96)	2/194	0.31 (0.06, 1.57)	37/434	1.49 (0.98, 2.27)	33/434	1.17 (0.76, 1.80)
≥5	8/160	0.53 (0.24, 1.17)	18/160	1.22 (0.70, 2.15)	3/160	0.51 (0.11, 2.39)	4/160	0.51 (0.14, 1.80)	11/320	0.53 (0.26, 1.06)	22/320	1.06 (0.63, 1.77)
**6–10-year exposure window**												
**No vs. any dispensation**												
0	26/331	1.00	34/331	1.00	5/267	1.00	6/267	1.00	31/598	1.00	40/598	1.00
≥1	50/528	1.43 (0.83, 2.45)	78/528	1.54 (0.98, 2.43)	9/458	0.86 (0.25, 2.97)	9/458	0.70 (0.22, 2.25)	59/986	1.32 (0.80, 2.16)	87/986	1.39 (0.91, 2.12)
**Number of dispensations**												
0	26/331	1.00	34/331	1.00	5/267	1.00	6/267	1.00	31/598	1.00	40/598	1.00
1–2	37/329	1.79 (1.01, 3.20)	48/329	1.57 (0.95, 2.57)	4/259	0.75 (0.17, 3.37)	5/259	0.82 (0.23, 2.98)	41/588	1.60 (0.93, 2.74)	53/588	1.44 (0.91, 2.29)
3–4	9/120	0.93 (0.39, 2.21)	19/120	1.45 (0.76, 2.77)	2/105	0.79 (0.13, 4.66)	2/105	0.55 (0.09, 3.26)	11/225	0.90 (0.41, 1.97)	21/225	1.30 (0.71, 2.38)
≥5	4/79	0.77 (0.25, 2.43)	11/79	1.61 (0.75, 3.48)	3/94	1.17 (0.21, 6.51)	2/94	0.60 (0.10, 3.59)	7/173	0.87 (0.34, 2.26)	13/173	1.38 (0.68, 2.80)

Model adjusted for age, sex, BMI, smoking, physical activity, education, and family history of diabetes.
$GADA_{low}$ – GAD autoantibody levels <median, $GADA_{high}$ – GAD autoantibody levels ≥median.
*A small proportion classified as LADA cases in ANDIS did not consent to share clinical information with ESTRID (n=11). These cases do not contribute to the stratified analysis. Only cases with GADA from HUNT4 included.

ESM Table 10. Odds ratios and 95% confidence intervals for the association between exposure to **dermatologicals and the risk of LADA or type 2 diabetes**, within different exposure windows prior to diagnosis/matching. (ESTRID)

	LADA		Type 2 diabetes	
	**Cases/ controls (n)**	**aOR (95% CI)**	**Cases/ controls (n)**	**aOR (95% CI)**
**0–<1-year exposure window**				
**No vs. any dispensation**				
0	561/2275	1.00	1895/2275	1.00
≥1	36/111	1.23 (0.82, 1.85)	170/111	1.06 (0.63, 1.79)
**1–5-year exposure window**				
**No vs. any dispensation**				
0	427/2092	1.00	1621/2092	1.00
≥1	75/293	1.22 (0.91, 1.64)	296/293	1.12 (0.89, 1.39)
**Number of dispensations**				
0	427/2092	1.00	1621/2092	1.00
1–2	55/215	1.24 (0.88, 1.73)	208/215	1.12 (0.87, 1.44)
3–4	12/35	1.80 (0.88, 3.66)	45/35	1.68 (0.95, 2.95)
≥5	8/43	0.76 (0.34, 1.70)	43/43	0.74 (0.42, 1.29)
Per dispensation	502/2385	1.00 (0.93, 1.08)	1917/2385	0.99 (0.94, 1.05)
**6–10-year exposure window**				
**No vs. any dispensation**				
0	155/728	1.00	601/728	1.00
≥1	38/131	1.27 (0.82, 1.97)	126/131	1.02 (0.73, 1.43)
**Number of dispensations**				
0	155/728	1.00	601/728	1.00
1–2	29/94	1.40 (0.85, 2.30)	86/94	0.97 (0.66, 1.44)
3–4	7/23	1.15 (0.46, 2.90)	23/23	1.12 (0.55, 2.28)
≥5	2/14	0.65 (0.13, 3.17)	17/14	1.17 (0.46, 2.96)
Per dispensation	193/859	1.04 (0.91, 1.19)	727/859	1.02 (0.93, 1.12)
**0–10-year exposure window**				
**No vs. any dispensation**				
0	136/657	1.00	521/657	1.00
≥1	57/202	1.38 (0.95, 2.02)	206/202	1.13 (0.86, 1.50)
**Number of dispensations**				
0	136/657	1.00	521/657	1.00
1–2	40/133	1.50 (0.97, 2.31)	125/133	1.05 (0.75, 1.47)
3–4	9/33	1.42 (0.62, 3.26)	35/33	1.49 (0.81, 2.74)
≥5	8/36	0.96 (0.41, 2.24)	46/36	1.13 (0.63, 2.02)
Per dispensation	193/859	1.00 (0.94, 1.07)	727/859	1.00 (0.95, 1.04)

Model adjusted for age, sex, BMI, smoking, physical activity, educational level, and family history of diabetes.

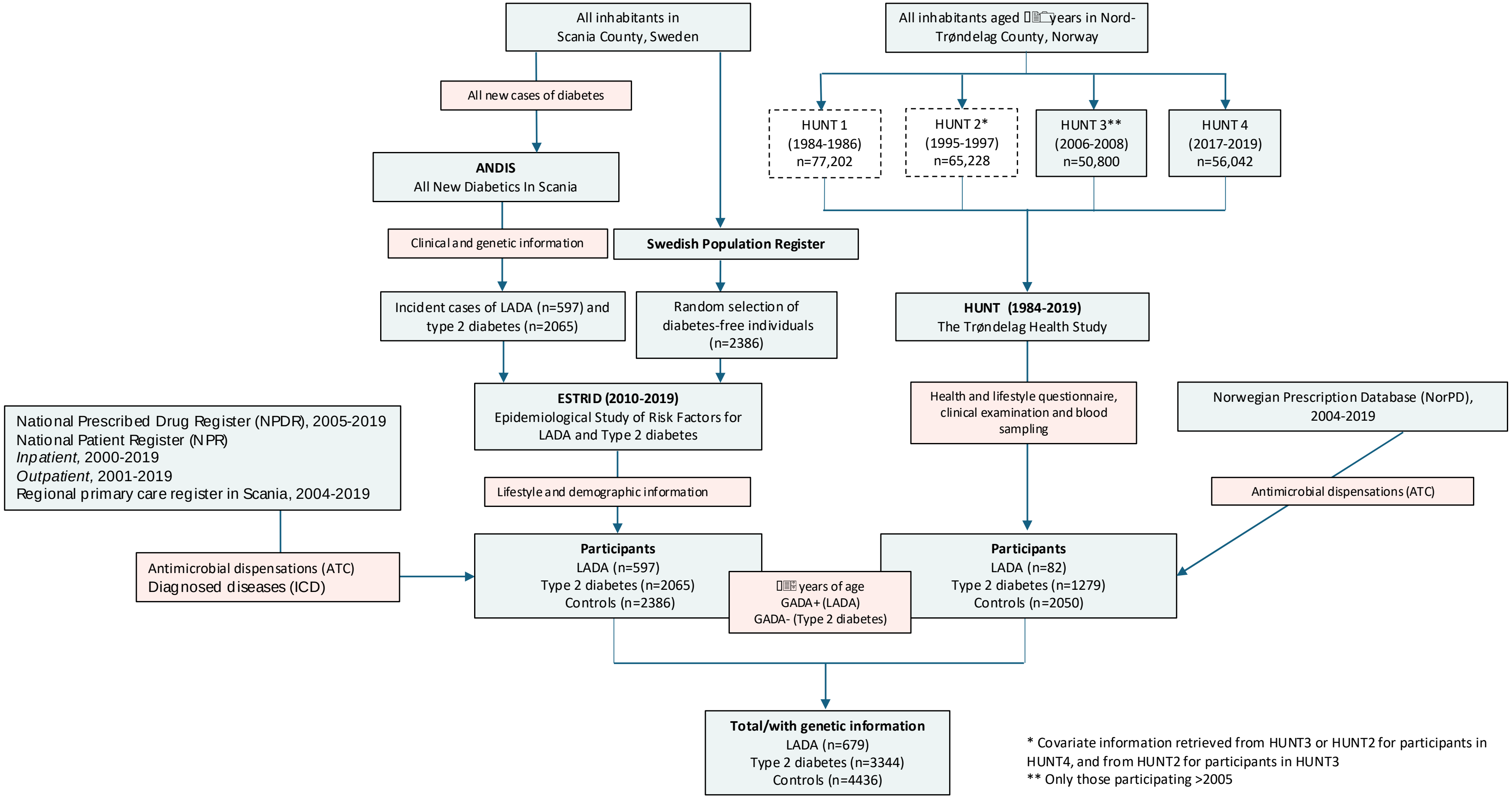

ESM Figure 1. Study design

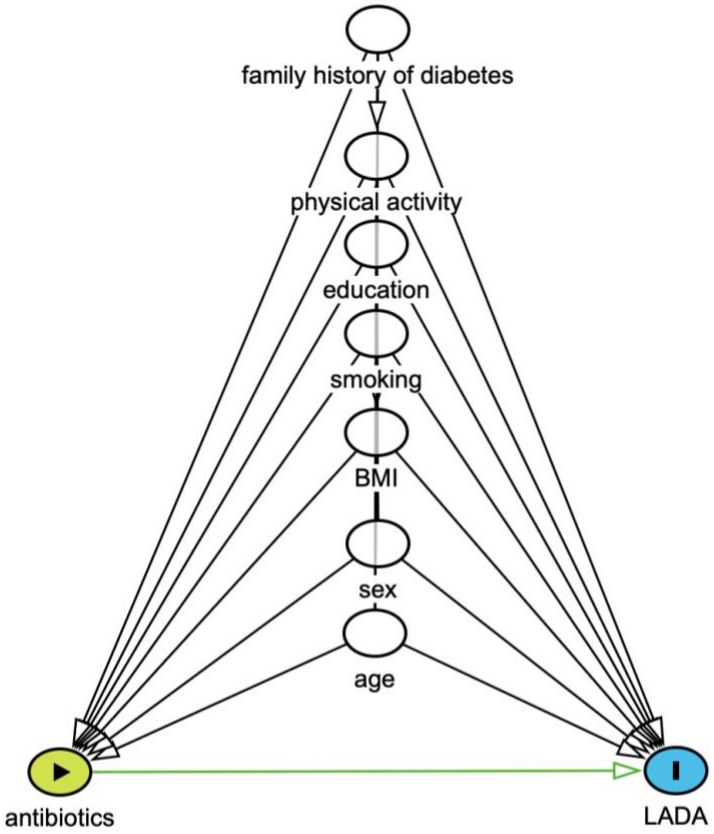

ESM Figure 2. Directed acyclic graph (DAG) representing the covariate selection in the fully adjusted model (Model 2). Non-essential paths are covered by the central line/arrows in the figure to improve readability. Each confounder is assumed to have an independent effect on the exposure and outcome.

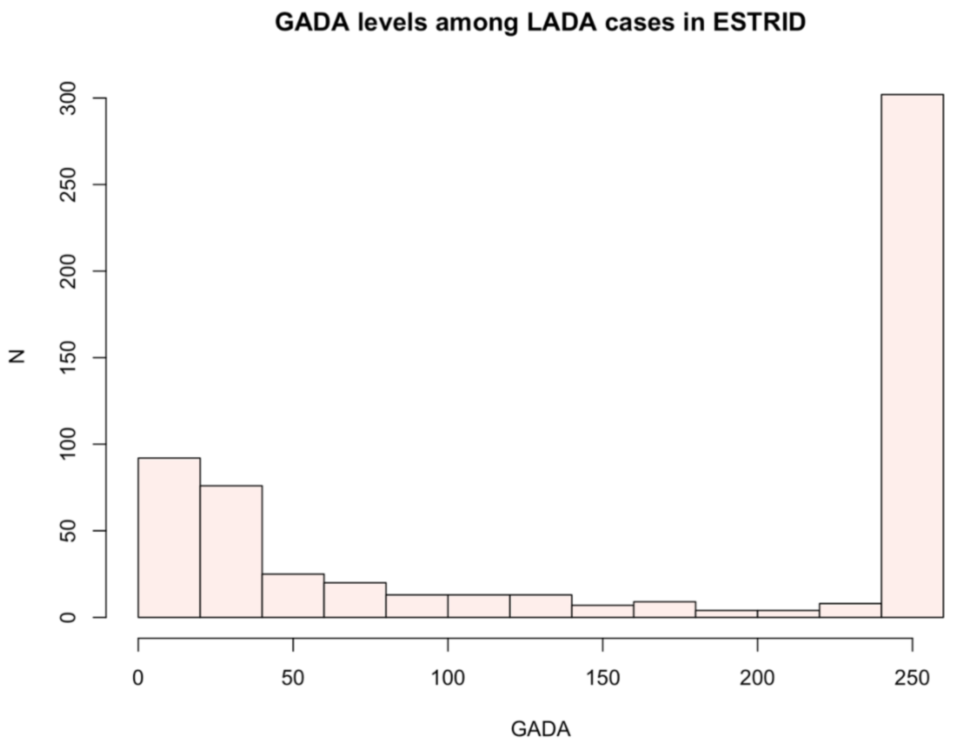

ESM Figure 3. Distribution of GADA levels among LADA cases in ESTRID.

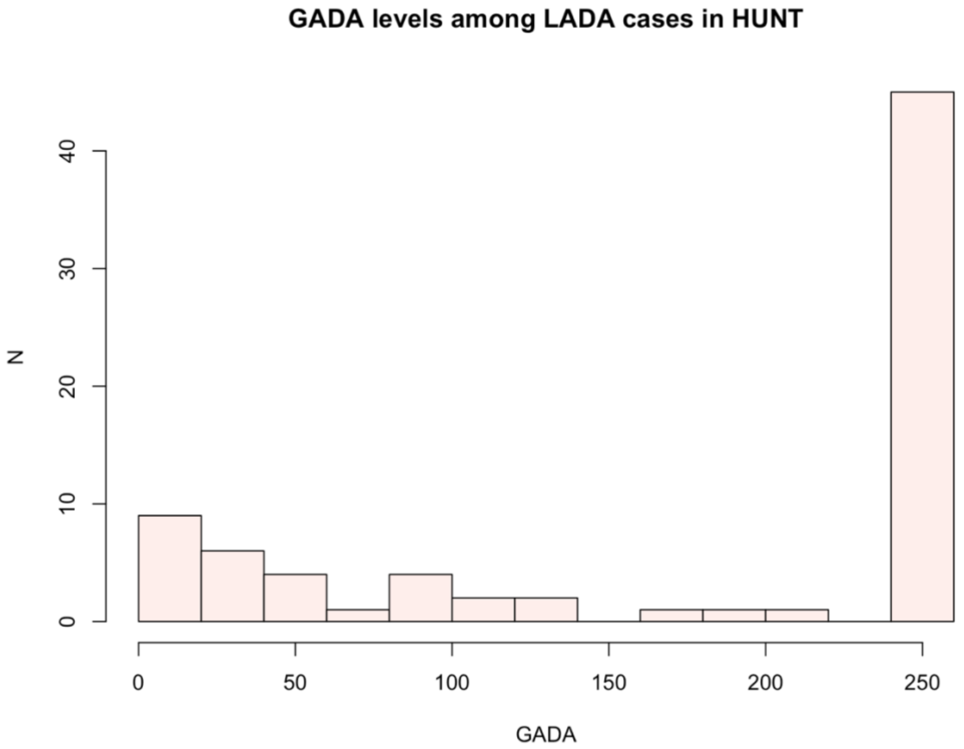

ESM Figure 4. Distribution of GADA levels among LADA cases in HUNT

References

1. Ahlqvist E, Storm P, Käräjämäki A, et al (2018) Novel subgroups of adult-onset diabetes and their association with outcomes: a data-driven cluster analysis of six variables. Lancet Diabetes Endocrinol 6(5):361–369. https://doi.org/10.1016/S2213-8587(18)30051-2

2. Vandenbroucke JP, Pearce N (2012) Case-control studies: Basic concepts. Int J Epidemiol 41(5):1480–1489. https://doi.org/10.1093/ije/dys147

3. Rassen J, Doherty M, Huang W, Schneeweiss S Pharmacoepidemiology Toolbox

4. Schneeweiss S, Rassen JA, Glynn RJ, Avorn J, Mogun H, Brookhart MA (2009) High-dimensional propensity score adjustment in studies of treatment effects using health care claims data. Epidemiology 20(4):512–522. https://doi.org/10.1097/EDE.0b013e3181a663cc

5. Rassen JA, Glynn RJ, Brookhart MA, Schneeweiss S (2011) Covariate selection in high-dimensional propensity score analyses of treatment effects in small samples. Am J Epidemiol 173(12):1404–1413. https://doi.org/10.1093/aje/kwr001
